# Competitive Fitness of Essential Gene Knockdowns Reveals a Broad-Spectrum Antibacterial Inhibitor of the Cell Division Protein FtsZ

**DOI:** 10.1128/AAC.01231-18

**Published:** 2018-11-26

**Authors:** Andrew M. Hogan, Viola C. Scoffone, Vadim Makarov, April S. Gislason, Haben Tesfu, Maria S. Stietz, Ann Karen C. Brassinga, Michael Domaratzki, Xuan Li, Alberto Azzalin, Marco Biggiogera, Olga Riabova, Natalia Monakhova, Laurent R. Chiarelli, Giovanna Riccardi, Silvia Buroni, Silvia T. Cardona

**Affiliations:** a Department of Microbiology, University of Manitoba, Winnipeg, Manitoba, Canada; b Department of Biology and Biotechnology, University of Pavia, Pavia, Italy; c Federal Research Center Fundamentals of Biotechnology of the Russian Academy of Sciences, Moscow, Russia; d Department of Computer Science, University of Manitoba, Winnipeg, Manitoba, Canada; e Department of Mathematics and Statistics, University of Minnesota-Duluth, Duluth, Minnesota, USA; f Department of Internal Medicine and Therapeutics, University of Pavia, Fondazione IRCCS San Matteo, Pavia, Italy; g Department of Medical Microbiology and Infectious Disease, University of Manitoba, Winnipeg, Canada

**Keywords:** *Burkholderia*, FtsZ, Tn-seq, drug targets, essential genes, fluorescent image analysis, mechanisms of action

## Abstract

To streamline the elucidation of antibacterial compounds’ mechanism of action, comprehensive high-throughput assays interrogating multiple putative targets are necessary. However, current chemogenomic approaches for antibiotic target identification have not fully utilized the multiplexing potential of next-generation sequencing. Here, we used Illumina sequencing of transposon insertions to track the competitive fitness of a Burkholderia cenocepacia library containing essential gene knockdowns. Using this method, we characterized a novel benzothiadiazole derivative, 10126109 (C109), with antibacterial activity against B. cenocepacia, for which whole-genome sequencing of low-frequency spontaneous drug-resistant mutants had failed to identify the drug target. By combining the identification of hypersusceptible mutants and morphology screening, we show that C109 targets cell division. Furthermore, fluorescence microscopy of bacteria harboring green fluorescent protein (GFP) cell division protein fusions revealed that C109 prevents divisome formation by altering the localization of the essential cell division protein FtsZ. In agreement with this, C109 inhibited both the GTPase and polymerization activities of purified B. cenocepacia FtsZ. C109 displayed antibacterial activity against Gram-positive and Gram-negative cystic fibrosis pathogens, including Mycobacterium abscessus. C109 effectively cleared B. cenocepacia infection in the Caenorhabditis elegans model and exhibited additive interactions with clinically relevant antibiotics. Hence, C109 is an enticing candidate for further drug development.

## Abstract (precis)

To streamline the elucidation of antibacterial compounds’ mechanism of action, comprehensive high-throughput assays interrogating multiple putative targets are necessary. However, current chemogenomic approaches for antibiotic target identification have not fully utilized the multiplexing potential of next-generation sequencing.

## INTRODUCTION

Imperative to the fight against antibiotic resistance, new antibiotic discovery strategies and platforms must be employed. These platforms must provide rapid target and mechanism-of-action identification, as these are recognized as challenging aspects of antimicrobial screening (1). Many versions of target-based assays now exist which examine the effect of antimicrobials on specific targets (1). In order to vastly increase throughput and the number of simultaneous targets examined, next-generation sequencing (NGS) can be coupled to target-based assays in whole cells (2–4). The power of these NGS hybrid assays lies in the ability to profile the specific abundances of mutants within genomic libraries with high sensitivity and multiplexing potential (3). While the development of these platforms in yeast has been fruitful (5–7), the potential of NGS to match new antimicrobials to their targets in bacteria is still emerging.

Burkholderia cenocepacia is a member of the Burkholderia cepacia complex (Bcc), a group of at least 20 opportunistic human pathogens (8). Immunocompromised hosts, such as those with cystic fibrosis, are particularly susceptible to B. cenocepacia infections. For cystic fibrosis patients, respiratory infection is the leading cause of mortality. Members of the Bcc are almost completely resistant to aminoglycosides, cationic peptides, and β-lactams (9). Furthermore, the resistance of Bcc isolates can increase over time during successive pulmonary exacerbations (10). As a consequence of the lack of effective antibiotics, Bcc infections are difficult to eradicate and can result in cepacia syndrome, a lethal form of pneumonia (11). We have shown that the novel synthetic methyl [(4-nitro-2,1,3-benzothiadiazol-5-yl)thio]acetate 10126109 (here called C109) is a bactericidal antimicrobial against Bcc species (12). However, the mechanism of action and target of C109 are unknown, warranting further investigations. Here, we employed a combination of an NGS-based fitness assay, fluorescence and electron microscopy, and biochemical assays to show that C109 is a broad-spectrum antibacterial that inhibits the cell division protein FtsZ, an attractive target for antibiotic discovery (13). Opposite of most FtsZ inhibitors, C109 is active against Gram-negative bacteria and shows properties that merit its development as a new antibacterial drug.

## RESULTS

### An Illumina-based fitness assay reveals mutants hypersusceptible to C109.

Previously, we developed a high-density transposon mutant (HDTM) library in B. cenocepacia K56-2 by delivering a transposon element containing an outward rhamnose-inducible promoter (*P_r_*_haB_) into the genome (14). Using an enrichment process to isolate mutants in which *P_r_*_haB_ is driving the expression of essential genes, we built a redundant knockdown library of 830 clones. This library was combined with another 134 previously constructed knockdown mutants (15), in total representing 83 essential operons. The combined knockdown mutant library showed good representation of Cluster of Orthologous Groups (COG) categories compared to the essential genome of B. cenocepacia K56-2 (see Fig. S1 in the supplemental material).

**FIG 1:**
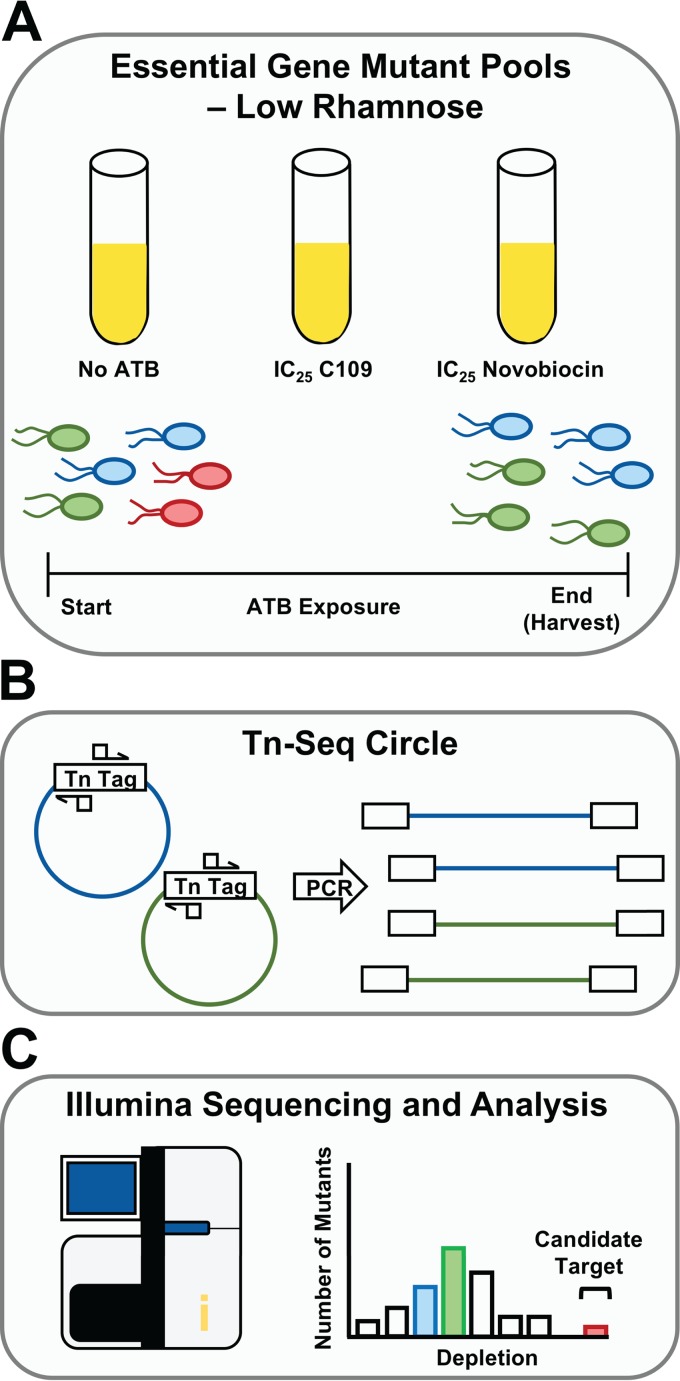
Workflow of competitive fitness assay. (A) The sensitized mutant library is grown competitively without antibiotics (ATB; control), or with the IC_25_ of C109 or novobiocin. Growth with antibiotics selectively depletes certain mutants. (B) To track mutant abundance, transposon-genome junctions are enriched using Tn-Seq Circle. (C) Samples are sequenced on a HiSeq platform, and reads are then mapped to the *B. cenocepacia* K56-2 genome to call insertion sites. The reads from the antimicrobial-treated conditions are compared to the no-antibiotic controls to determine highly depleted mutants, which are used to call candidate antibacterial-target matches.

**FIG 2:**
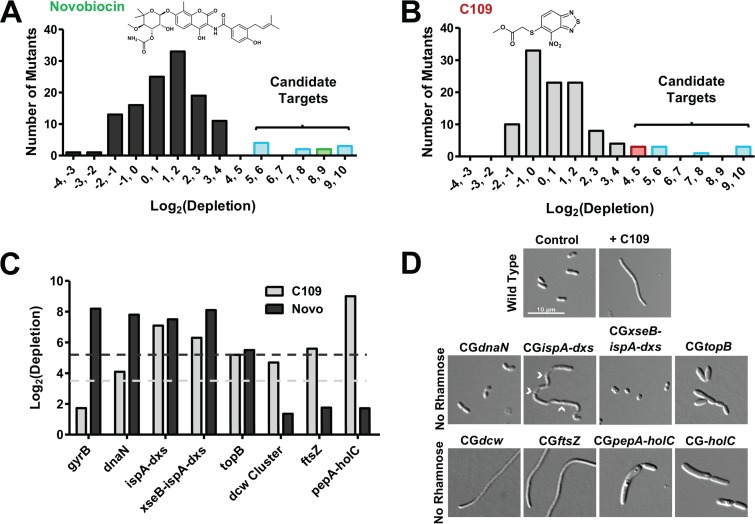
A Tn-seq-based fitness assay and morphological phenotype screening links C109 to the *dcw* operon. (A and B) Hypersusceptibility of knockdown mutants to novobiocin (Novo) (A) and C109 (B). Only mutants with depletion ratios of a *P* value of <0.05 are shown. The region highlighted as “candidate targets” corresponds to knockdown mutants with log_2_ (depletion) greater than two standard deviations from the mean. (C) Comparison of hypersusceptible mutants to C109 and novobiocin. Light gray and charcoal dashes represent 2-standard deviation (2-SD) thresholds for novobiocin and C109, respectively. (D) Morphology of *B. cenocepacia* and knockdown mutants treated with 2× the MIC (16 μg/ml) C109 or incubated without rhamnose, respectively, for 6 h. Chevrons indicate septa in CG*ispA-dxs*. All micrographs are to the same scale.

To investigate the mechanism of action of C109, we developed the experimental approach shown in Fig. 1. Briefly, when the combined knockdown mutant library is pooled and grown under sensitizing conditions (low rhamnose) in the presence of an antibacterial molecule, hypersusceptible mutants become depleted. The relative abundances of the mutants after antibiotic treatment are detected by Illumina sequencing of the transposon-genome junctions (transposon sequencing [Tn-seq]) (16, 17). As previously observed (2, 15), when mutant pools were exposed to the 25% inhibitory concentration (IC_25_) of novobiocin, a mutant with a knockdown in the target coding gene *gyrB* (locus tag WQ49_RS23250) was highly depleted (Fig. 2A and C). In addition, knockdown mutants in four transcriptional units were hypersusceptible to both novobiocin and C109 (*dnaN*, *xseB-ispA-dxs*, *ispA-dxs*, and *topB*) (Fig. 2A to C). A fifth hypersusceptible knockdown mutant of *lolB* (WQ49_RS25155) was removed from further analysis, as only one of several mutants in this gene (Table S1) was hypersusceptible to novobiocin and C109. Closer inspection revealed that the transposon insertion in the only hypersusceptible mutant truncated the first 15 residues from LolB, which is likely the secretory signal peptide (18), resulting in reduced viability. Knockdown mutants of two essential operons containing cytosol aminopeptidase (*pepA*, WQ49_RS26825) and DNA polymerase III subunit chi (*holC*, WQ49_RS26830), and another containing the division and cell wall cluster (*dcw*, WQ49_RS00040 to WQ49_RS00110), which includes *ftsZ*, were hypersusceptible to C109 but not to novobiocin (Fig. 2A to C).

Chemical and genetic depletion of essential gene function affects cell morphology (19). As such, we reasoned that knockdown of the target of C109 might phenocopy the effect of C109 treatment, further narrowing the list of putative targets. When grown without rhamnose, the mutants hypersusceptible to C109 displayed a variety of morphologies (Fig. 2D). To facilitate comparison, we developed a qualitative characteristic matrix based on filamentation, enlargement, shortening, and bending (Table S2). Compared to the wild-type morphology, C109 treatment caused a marked filamentous phenotype that was also observed upon knockdown of the isoprenoid synthesis *ispA* and *dxs*, and *dcw* cluster genes, including knockdown of *ftsZ* (Fig. 2D). However, filaments formed by *ispA* and *dxs* knockdown were septated and severely bent, a phenotype not observed in C109-treated cells. Additionally, the knockdowns of the other candidate targets caused enlargement on both the lateral and longitudinal cell axes, or cell shortening, but not filamentation (Fig. 2D and Table S2). Together, the hypersusceptibility of the *dcw* knockdown to C109 and the similar morphologies of the C109-treated cells and the *dcw* knockdowns suggest that C109 inhibits a function encoded by the *dcw* cluster.

### C109 inhibits divisome formation and induces filamentation.

The *dcw* cluster is a well-conserved group of genes encoding functions centered on cell wall synthesis and cell division (20). Included are proteins required to form the divisome, a structure whose orderly assembly, beginning with FtsA and FtsZ, is critical for cell division (21). Perturbation of the recruitment timing or localization of divisome proteins prevents cell division and causes filamentation (22).

**FIG 3:**
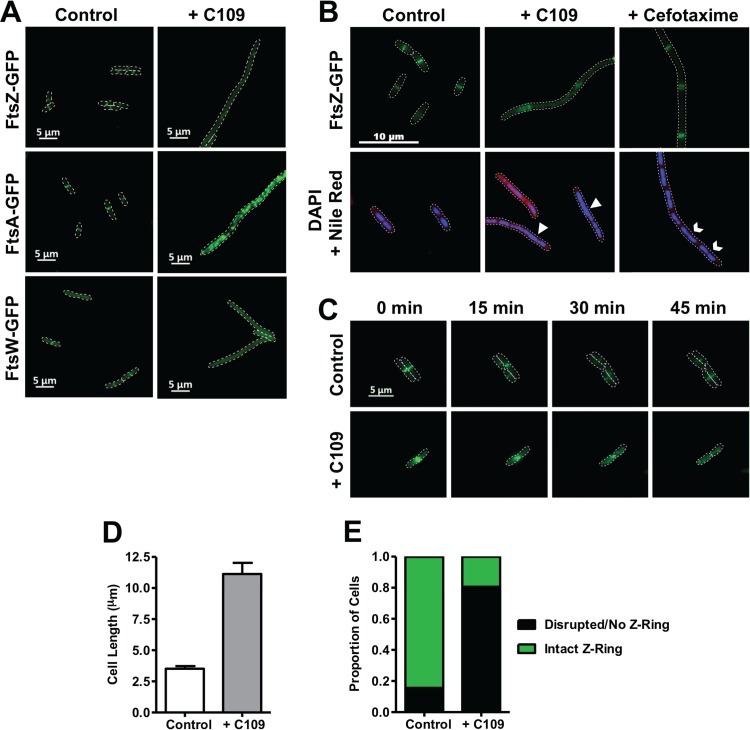
C109 causes cell filamentation and disrupts divisome formation. (A and B) Localization of FtsZ (A and B), FtsA (A), and FtsW (A) upon 3-h treatment of exponentially growing ASKA strains with C109 (A and B) or cefotaxime (B). Arrowheads show areas with deficient nucleoid segregation. Chevrons show proper nucleoid segregation. (C) Time-lapse fluorescence microscopy of FtsZ-GFP cells, spotted onto an agarose pad with or without 4 μg/ml C109. Dashes denote cell boundaries. (D and E) Cell length (D) and proportion of cells with Z-ring (E) after 3 h of exposure to C109 (*n* = 200 cells).

To systematically assess if C109 prevents the proper localization of divisome components, we used a subset of the ASKA collection of Escherichia coli strains expressing C-terminal green fluorescent protein (GFP) fusions of all *dcw* cluster genes (23). E. coli is a valid tool for use with C109, as its growth is also inhibited by C109 (Table 1), and the organizations of the *dcw* clusters of E. coli and B. cenocepacia are nearly identical (20, 24). In the absence of C109, FtsZ-GFP, FtsA-GFP, and FtsW-GFP localized to the midcell, as expected (Fig. 3A). MraZ-GFP also appeared to localize correctly, in the nucleoid region (Fig. S2). Upon treatment with C109, the localization of the transcription factor MraZ-GFP did not markedly change; however, the fluorescence corresponding to the central divisome components FtsZ-GFP and FtsA-GFP appeared as dispersed puncta, or as in the case of FtsW-GFP, became diffuse (Fig. 3A). Other divisome protein-GFP fusions localized properly with or without C109 (Fig. S2A). FtsI-GFP, FtsL-GFP, MraY-GFP, and FtsQ-GFP did not properly localize to the midcell under control conditions (Fig. S2B) and were excluded from further testing.

**TABLE 1:** Antibacterial activity of C109 against select Gram-negative and Gram-positive bacteria

Strain	Features	MIC (µg/ml)^*a*^	Source
Acinetobacter baumannii ATCC 19606	Reference strain	16	ATCC
Burkholderia cenocepacia K56-2	ET12 lineage cystic fibrosis clinical isolate	8	73
Burkholderia cenocepacia J2315	ET12 lineage cystic fibrosis clinical isolate	8	74
Enterobacter aerogenes ENT001	Urine meropenem-resistant isolate	32	A. Kumar
*E*. aerogenes ATCC 13048	Reference strain	8	A. Kumar
Escherichia coli ATCC 25922	Reference strain	8	ATCC
E. coli 117782	ESBL-positive clinical isolate	4	G. Zhanel
E. coli 120955	ESBL-positive clinical isolate	8	G. Zhanel
Klebsiella pneumoniae 119178	ESBL-positive clinical isolate	32	G. Zhanel
Mycobacterium abscessus 13NC740779	Ciprofloxacin-resistant clinical isolate	4	H. Adam
M. abscessus 14NF251095	Ciprofloxacin-resistant clinical isolate	4	H. Adam
M. abscessus 14NJ168168	Ciprofloxacin-resistant clinical isolate	4	H. Adam
M. abscessus 16NH386647	Ciprofloxacin-resistant clinical isolate	8	H. Adam
Pseudomonas aeruginosa PAO1	Common lab strain, from burn wound	256	A. Kumar
P. aeruginosa PA7	Nonrespiratory clinical isolate	128	A. Kumar (75)
P. aeruginosa PA14	High virulence burn wound isolate	>128	Joseph Lam (76)
Salmonella enterica serovar Typhimurium	SGI1 genomic island; multidrug resistant	64	77
Serratia marcescens Db11	Streptomycin-resistant strain	16	78
Staphylococcus aureus ATCC 25923	Methicillin-sensitive reference strain	4	ATCC

a MIC values are given as the median of three biological replicates.

Filamentation is a consequence of cell division inhibition (22), yet other cellular processes not related to cell division can cause filamentation, which in turn can prevent divisome formation (25–27). We therefore assessed the possibility that the observed filamentation caused by C109 is not directly associated with inhibiting divisome formation. We first examined the localization of FtsZ-GFP as a marker for early divisome formation in response to cefotaxime, a β-lactam cell wall synthesis inhibitor known to cause filamentation (28). In the presence of cefotaxime, the E. coli cells had filamentous morphology, yet FtsZ-GFP was regularly localized in bands along the filament (Fig. 3B), showing that cell filamentation can be uncoupled from Z-ring formation. Conversely, the filamentous cells formed due to C109 treatment showed mislocalization of FtsZ-GFP, reinforcing the view that C109 specifically targets divisome formation. Moreover, treatment with C109 appeared to cause nucleoid segregation deficiency, which can be a consequence of inhibited divisome assembly (29, 30). As opposed to cells treated with C109, those treated with cefotaxime showed defined nucleoids along the filament (Fig. 3B). To further demonstrate that C109 inhibits divisome assembly, we examined how C109 affects the distribution of FtsZ-GFP in live cells using fluorescence time-lapse microscopy. FtsZ-GFP rapidly localized to the midcell site in untreated cells (Fig. 3C), while in cells treated with C109, FtsZ-GFP did not localize properly but instead formed puncta throughout the cell. Importantly, we observed that inhibition of FtsZ-GFP localization occurred before filamentation, further suggesting that C109-induced divisome inhibition causes filamentation. After only 3 h of exposure to 1× the MIC of C109, we observed a 3-fold increase in length on average (Fig. 3D), and 80% of cells had an absent or disrupted Z-ring, compared to 20% in the control (Fig. 3E). Together, these findings support the notion that C109 blocks cell division by inhibiting divisome formation, which in turn causes a filamentous morphology.

### Genetic evidence suggests that FtsZ is the target of C109.

**FIG 4:**
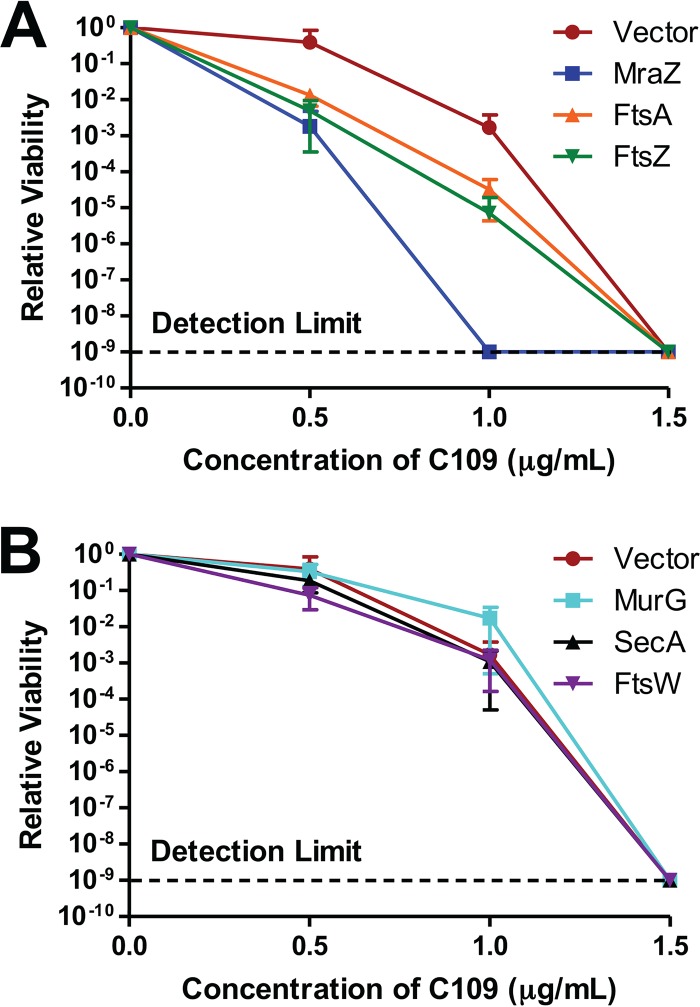
Mild overexpression of MraZ-GFP, FtsA-GFP, and FtsZ-GFP but not FtsW-GFP, MurG-GFP, or SecA-GFP sensitizes cells to C109. Cells were induced with 10 µM IPTG and then exposed to C109 for 12 h. A spot plate was used to count the CFU per milliliter. Counts are relative to each strain without C109 treatment. (A) Growth of strains expressing MraZ-GFP, FtsA-GFP, and FtsZ-GFP. (B) Growth of strains expressing MurG-GFP, SecA-GFP, and FtsW-GFP. Vector refers to the strains harboring pCA24N-empty. Error bars show mean ± SD, with *n* = 3 biological replicates.

Target overexpression is known to decrease susceptibility to certain antibiotics (31). To seek additional evidence that the target of C109 is encoded by the *dcw* cluster, we mildly overexpressed MraZ-GFP, MurG-GFP, FtsW-GFP, FtsA-GFP, and FtsZ-GFP, and SecA-GFP from an isopropyl thio-β-d-galactopyranoside (IPTG)-inducible promoter in E. coli. These fusions were chosen because they localized correctly without C109. SecA-GFP was used as a control, as it is not encoded by the *dcw* cluster. The concentration of IPTG was first titrated to not cause a growth defect in any of the strains (Fig. S3); hence, 10 μM IPTG was selected. As shown in Fig. 4A, the strains expressing MraZ-GFP, FtsA-GFP, and FtsZ-GF had a higher susceptibility to C109 than the control strain. On the contrary, the strains expressing SecA-GFP, FtsW-GFP, and MurG-GFP displayed a susceptibility to C109 similar to that of the control (Fig. 4B). The higher susceptibility of the strain expressing MraZ-GFP was expected, since overexpression of MraZ is known to perturb cell division, causing a lethal effect (32). As the proper localization of FtsZ and FtsA was affected by C109, we were expecting that increased expression of these proteins might permit continued cell division, reducing susceptibility to C109. However, the increased susceptibility suggests toxic interactions between C109, FtsA, and FtsZ. Toxic antibacterial-target interactions have been reported previously (31). These results, together with the sequential assembly of the divisome as a logic model means that the most upstream protein with C109-inhibited localization could be the target. Therefore, our results suggest that the target of C109 is FtsZ.

**FIG 5:**
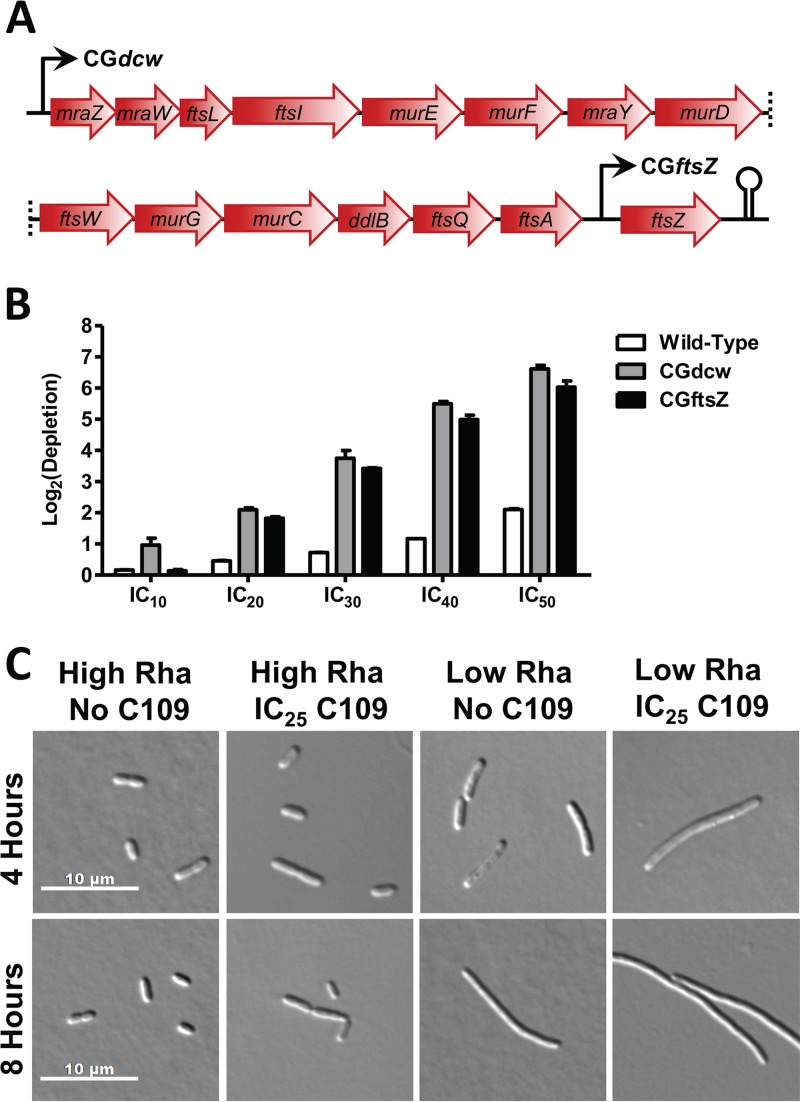
Knockdown of *ftsZ* sensitizes cells to C109. (A) Organization of the B. cenocepacia *dcw* cluster and positions of the rhamnose (Rha)-inducible promoters in CG*dcw* and CG*ftsZ*. (B) Hypersusceptibility of sensitized B. cenocepacia knockdown mutants grown clonally in response to increasing concentrations of C109. Error bars show mean ± SD, with *n* = 3 biological replicates. (C) Morphology of CG*ftsZ* with high (0.20%) or low (0.04%) rhamnose, with or without C109.

To further support the idea that FtsZ is the *in vivo* target of C109, we used homologous recombination to create a B. cenocepacia mutant with rhamnose-inducible control of *ftsZ* expression (CG*ftsZ*) (Fig. 5A). If FtsZ is targeted by C109, one would expect that knockdown of *ftsZ* expression would cause increased susceptibility to C109. When grown under low-rhamnose conditions, we observed that CG*ftsZ* and CG*dcw* were both hypersusceptible to C109 compared to the wild-type control (Fig. 5B). Importantly, CG*ftsZ* and CG*dcw* were susceptible to very similar levels, suggesting that the entire susceptibility phenotype of the CG*dcw* mutant can be reproduced by knocking down *ftsZ* alone. These results are further corroborated by the specific depletion of CG*ftsZ* during competitive growth in the presence of C109 (Fig. 2C) and by a filamentous morphology that resembles that of wild-type cells treated with C109 (Fig. 2D). Further, the filamentous phenotype of CG*ftsZ* was exacerbated in the presence of C109 (Fig. 5C and S4). We found that CG*ftsZ* was not susceptible to novobiocin but displayed susceptibility to C109 comparable to those mutants with knockdown of the *dcw* cluster (Fig. 2C). Examination of the filaments showed the presence of multiple nucleoids (Fig. S4B), and we propose this is why CG*dcw* and CG*ftsZ* were not the most highly depleted mutants in our fitness assay. Tn-seq detects copies of transposon-genome junctions, and we suggest that the multinucleoid phenotype may have partially masked the susceptibility of these cells to C109, reducing the specificity of our assay.

### C109 targets critical functions of FtsZ.

**FIG 6:**
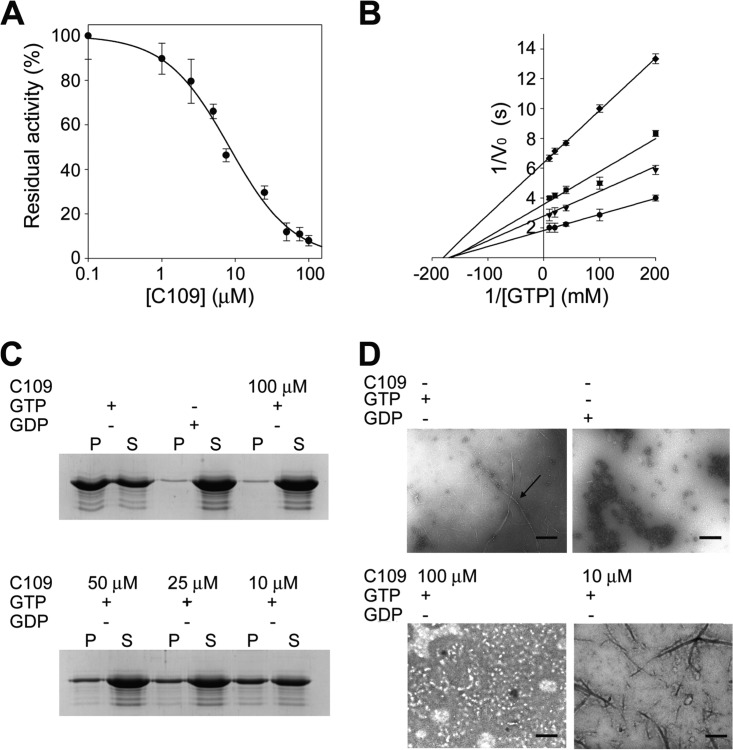
GTPase activity and polymerization assays demonstrate that C109 is a noncompetitive inhibitor of FtsZ. (A) IC_50_ determination of C109 against BcFtsZ. (B) Kinetic analyses of BcFtsZ in the presence of different C109 concentrations ranging from 0 to 50 μM. (C) SDS-PAGE of the sedimentation assay in the presence of different C109 concentrations. (D) Structure of FtsZ polymers in the presence of C109 (indicated by the arrow) visualized by TEM. Scale bar = 0.6 μm. Data are the mean ± SD of the results from three different replicates; images are representative of at least three different experiments.

The biochemical functions of FtsZ are to hydrolyze GTP and polymerize, thereby directing septal peptidoglycan synthesis (33). Therefore, disruption of these activities might be the mechanism of action of C109. To definitively validate the target of C109 as FtsZ, we first assessed the effect of C109 on the *in vitro* GTPase activity of B. cenocepacia FtsZ. Recombinant FtsZ was purified to homogeneity in a soluble monomeric catalytically active form (Fig. S5). Using a coupled spectrophotometric assay, we demonstrated that the protein catalyzed the hydrolysis of GTP (*K_m_* = 6.4 ± 0.8 μM). Moreover, using a previously described sedimentation protocol (34) and negative-stain transmission electron microscopy (TEM), we found that the recombinant FtsZ could form polymers. The enzymatic assay revealed that C109 inhibits the FtsZ GTPase activity with a 50% inhibitory concentration (IC_50_) of 8.2 ± 1.3 µM (Fig. 6A). Kinetic analyses in the presence of increasing concentrations of C109 showed that C109 behaves as a noncompetitive inhibitor (Fig. 6B), as the *K_m_* values for GTP are not altered by the presence of the compound, with a reduction in *V*_max_. These data are consistent with the hypothesis that C109 binds to a different site from the GTP binding site, inhibiting the polymerization and, consequently, the formation of the full GTPase active-site pocket. To support this hypothesis, the polymerization assay performed in the presence of C109 demonstrated concentration-dependent inhibition of polymerization (Fig. 6C and D). Additionally, we observed that C109 acts additively with the FtsZ filament-stabilizing antimicrobial PC190723 (35) in both methicillin-resistant Staphylococcus aureus (MRSA) (CF 225) and methicillin-susceptible S. aureus (MSSA) (ATCC 29213) (fractional inhibitory concentration [FIC] index values, 0.625 and 0.75, respectively). While this additive interaction suggests that C109 could bind to the interdomain cleft as PC190723, it is not clear why PC190723, but not C109, stabilizes FtsZ polymerization.

### Therapeutic potential of C109.

To expand upon the utility of C109 as a prospective antibiotic, we explored the spectrum of its activity against a variety of Gram-positive and Gram-negative pathogens (Table 1). C109 inhibits the growth of extended-spectrum beta-lactamase (ESBL)-producing strains of Escherichia coli, methicillin-sensitive Staphylococcus aureus, Serratia marcescens, Acinetobacter baumannii, Enterobacter aerogenes, and Mycobacterium abscessus. However, C109 was not active against Pseudomonas aeruginosa and Klebsiella pneumoniae. When further tested against a panel of cystic fibrosis Bcc isolates, a range of MICs were observed, from 4 to 64 μg/ml (Table 2), similar to those from the respective reference strains (12). C109 was also effective at inhibiting biofilm formation of B. cenocepacia K56-2, with a minimum biofilm inhibitory concentration (MBIC) of 4 μg/ml, which was lower than the MIC for the same strain. C109 was less capable of eradicating K56-2 residing in established biofilms (minimum biofilm eradication concentration [MBEC], 32 μg/ml), but the 4-fold increase over the MIC was substantially less than those previously seen for other antibiotics on Burkholderia biofilms (36).

**TABLE 2:** Antibacterial activity of C109 against Bcc clinical isolates

Strain	MIC (µg/ml)^*a*^	Source
B. ambifaria BCC0478	8	E. Mahenthiralingam
B. ambifaria BCC0485	4	E. Mahenthiralingam
B. cenocepacia FCF 28	32	79
B. cenocepacia FCF 29	8	79
B. cenocepacia FCF 30	32	79
B. cenocepacia FCF 31	32	79
B. cenocepacia C5424	8	D. Speert
B. cenocepacia CEP511	16	D. Speert
B. cenocepacia BCC1202	32	E. Mahenthiralingam
B. cenocepacia BCC0076	8	E. Mahenthiralingam
B. cenocepacia BCC1119	32	E. Mahenthiralingam
B. cepacia BCC1381	16	E. Mahenthiralingam
B. contaminans 2221	16	P. Drevinek
B. contaminans 4278	16	P. Drevinek
B. dolosa BCC0305	32	E. Mahenthiralingam
B. gladioli BCC1710	4	E. Mahenthiralingam
B. gladioli BCC1623	2	E. Mahenthiralingam
B. gladioli BCC1620	4	E. Mahenthiralingam
B. multivorans BCC1379	8	E. Mahenthiralingam
B. multivorans BCC0710	64	E. Mahenthiralingam
B. multivorans FCF 5	32	79
B. multivorans FCF 6	32	79
B. multivorans FCF 7	64	79
B. multivorans FCF 8	64	79
B. multivorans FCF 9	64	79
B. multivorans FCF 10	32	79
B. multivorans FCF 11	8	79
B. multivorans 454	64	P. Drevinek
B. multivorans 6094	64	P. Drevinek
B. pyrrocinia BCC0735	16	E. Mahenthiralingam
B. stabilis BCC0608	8	E. Mahenthiralingam
B. stabilis 3819	16	P. Drevinek
B. stabilis 9693	16	P. Drevinek
B. stabilis 10870	32	P. Drevinek
B. vietnamiensis BCC0296	16	E. Mahenthiralingam

a Median values of three biological replicates are shown.

With the increasing prevalence of antibiotic resistance, combination therapies are emerging as a possible method to improve pharmacological efficacy against recalcitrant infections (37, 38). With this in mind, we tested the *in vitro* interaction of antibiotics used to treat Bcc infection in cystic fibrosis patients (39). C109 acted additively in K56-2 with all eight antibiotics tested from many chemical classes (tobramycin, trimethoprim, meropenem, ceftazidime, ciprofloxacin, chloramphenicol, doxycycline, and novobiocin) (Table 3). Similar activity was observed in strain J2315 (Table 3). Therefore, C109 may be able to decrease the effective dose of many different classes of antibiotics used to treat Bcc infections.

**TABLE 3:** C109 has additive effects with common antibiotics used against *B. cenocepacia*

Antibiotic	K56-2 MIC alone (μg/ml)	K56-2 FIC index with C109^*a*^	Interpretation	J2315 MIC alone (μg/ml)	J2315 FIC index with C109^*a*^	Interpretation
Meropenem	32	0.625	Additive	32	0.508	Additive
Piperacillin	8	0.625	Additive	256	0.560	Additive
Tobramycin	512	0.625	Additive	512	2	Additive
Ciprofloxacin	2	1	Additive	8	2	Additive
Ceftazidime	64	0.625	Additive	ND	ND	
Doxycycline	4	0.625	Additive	ND	ND	
Novobiocin	8	0.75	Additive	ND	ND	
Trimethoprim	8	0.75	Additive	512	2	Additive
Chloramphenicol	32	1	Additive	ND	ND	

a Reported is the median FIC index from three biological replicates. ND, not determined.

To investigate applications for C109, we next determined if C109 is effective at clearing B. cenocepacia K56-2 infection. We used the C. elegans liquid killing assay (40) in which B. cenocepacia produces an intestinal infection in C. elegans. After 2 days, the survival of B. cenocepacia-infected nematodes treated with C109 was similar to that of nematodes fed with the nonpathogenic E. coli OP50 or nematodes treated with trimethoprim (Fig. S6). Furthermore, we exposed uninfected C. elegans nematodes to high concentrations of C109 to assess toxicity. Even at 128 μg/ml C109, 100% of the nematodes survived after 24 h (SURV_100_) (40) and 79% survived after 6 days (Table S3). We therefore calculated that the nematodes were able to survive at least 16-fold the concentration required to eradicate B. cenocepacia *in vitro* (Surv_100_/MIC) (Table S3). In addition, we investigated if C109 induces hemolysis at higher concentrations. Due to solubility limits, the highest concentration assessed was 128 μg/ml, at which we observed negligible hemolysis levels of approximately 3% (Table S3). We also evaluated the toxicity of C109 on the 16HBE (wild-type bronchiolar epithelial) and CFBE41o- (CF bronchiolar epithelial cells, homozygous for the ΔF508 mutation in *C*FTR) cell lines. Only at 75 μM (21.4 μg/ml), well above the MIC against B. cenocepacia of 8 μg/ml, the viability of both cell lines was reduced to 50% (50% toxic concentration [TC_50_]) (Table S3). To rule out possible inhibitory effects of C109 on mammalian tubulin, which is homologous to FtsZ, we performed a tubulin polymerization assay with a range of C109 concentrations (10 to 100 µM) (Fig. S7). This assay is based on the principle that light is scattered by microtubules proportionally to the concentration of microtubule polymers (41, 42). The resulting polymerization curve is representative of the three phases of microtubule polymerization, namely nucleation, growth, and steady-state equilibrium (Fig. S7). Compounds that interact with tubulin will alter one or more of the characteristic phases of polymerization. As an example, the antimitotic drug paclitaxel was used as a control; at 10 μM final concentration, it eliminates the nucleation phase and enhances the *V*_max_ of the growth phase. Indeed, C109 was not able to interfere with tubulin polymerization even at the highest concentration tested (Fig. S7).

## DISCUSSION

To combat the emergence of antibiotic resistance, one key approach is to develop classes of antibiotics against new targets. High-throughput assays show promise in addressing the bottleneck of target identification (1). Here, we outline the development and application of a novel competitive fitness screen for antimicrobial-target pairing in the antibiotic-resistant pathogen B. cenocepacia. Importantly, we identified that C109, a broad-spectrum antibacterial, inhibits the critical activities of FtsZ, thereby preventing cell division. Others have used gene knockdowns to identify antimicrobial targets (5, 43, 44); however, this is the first report to combine this approach with next-generation sequencing and a library enriched in essential gene mutants which represent many putative antibacterial targets. Furthermore, the competitive growth conditions provide an additional edge, that of magnified sensitivity versus clonal growth (2). However, our assay would unlikely be able to specifically match an antimicrobial that inhibits a nonprotein target. This is a known limitation of genetic screens; however, careful interpretation of the data may reveal the overall mechanism of action from disrupted genetic networks (5). During submission of this work, a study using Tn-seq with gene upregulation and machine learning was published (45). These authors demonstrated that lipid II is the target of the lysocins in S. aureus, therefore demonstrating that improvements in computational analysis can solve many of the challenges of chemogenomics.

Currently, there are no approved antibiotics that target FtsZ. It is, however, an attractive drug target because FtsZ is (i) essential for bacterial life, (ii) widely conserved across bacterial pathogens (iii) notably absent in mitochondria of higher eukaryotes, and (iv) evolutionarily distant from its eukaryotic counterpart, tubulin (46). Despite the broad conservation of FtsZ, there are few reported inhibitors with antimicrobial activity against Gram negatives (47–49), likely owing to the intrinsically lower permeability of the Gram-negative cell envelope (50). C109 is active against Gram positives, Gram negatives, and M. abscessus, suggesting that it is generally membrane permeable, perhaps due to its small size and cLogP value of 2.5. Notably, no spontaneous target-related mutations could be isolated upon exposure of B. cenocepacia to C109, suggesting that target-related resistance may not develop during therapeutic use of this drug.

A potential limitation of C109 for further development as an antibiotic could be its poor aqueous solubility. None of the C109 derivatives synthesized so far have shown improved solubility in water (data not shown), which may limit its utility for systemic administration. However, C109 is still an attractive drug for the development of an inhaled therapy to treat bacterial infections of the respiratory airways, where systemically delivered antimicrobials have been observed to have poor penetration (51). Even more, hypoxia followed by pulmonary vasoconstriction and chemokine-induced inflammation observed during pneumonia severely decrease the amount of drug delivered to the lung parenchyma (52). On the contrary, lipophilic antimicrobials directly deposited into the lungs by inhaled therapy should be slow to be absorbed into the systemic circulation, remaining in the lungs at higher concentrations while minimizing toxicity due to systemic exposure (53). In cystic fibrosis patients, where infections of the lower respiratory tract are particularly difficult to treat with systemically delivered antimicrobials, aerosolized antibacterial therapies are becoming more common, and the development of novel formulations for pulmonary delivery of more active drugs is an area of intense research (54).

FtsZ inhibitors could be applied in combination with approved antibiotics to improve potency. Alone, the inhibitor PC190723 is highly active *in vitro* against MRSA and linezolid-resistant and vancomycin-resistant S. aureus (35). PC190723 was found to synergize with seven diverse β-lactams and restore the sensitivity of MRSA strains to β-lactams (35). This synergy is based on the observation that PC190723 also inhibits the localization of penicillin-binding protein 2 (PBP2) (as it depends on FtsZ), which is responsible for septal peptidoglycan synthesis. It is suggested that the β-lactams then inactivate any residual PBP2 at the divisome (35). *In vitro* synergy was validated using a mouse model of MRSA infection, wherein PC190723 synergized with imipenem to strongly reduce bacterial load (35). Furthermore, in a screen against several β-lactams, those that inhibited PBP2 displayed the strongest synergy with TXA707, a prodrug form of PC190723 (55). We found that C109 did not synergize with any antibiotics currently prescribed for the treatment of Bcc infection (Table 3); however, the additive interaction with several antibiotics, such as meropenem and ceftazidime, would likely lower the effective dose required to clear Bcc infection. B. cenocepacia contains the genes encoding the PenA and AmpC β-lactamases, which are shown to give broad-spectrum resistance to many β-lactams, including imipenem and ceftazidime (56–58). We therefore suggest that the high levels of intrinsic resistance to β-lactams by B. cenocepacia could have masked synergistic interactions of C109 with β-lactams.

In summary, we outline the application and validation of a novel fitness screen to identify antimicrobial-target pairs in the cystic fibrosis pathogen B. cenocepacia. We propose that this method will allow the screening of many compounds in rapid succession against B. cenocepacia. Of further importance, the small size and hydrophobic characteristics of C109, in addition to additive interactions with other antibiotics, make this compound appropriate for inhaled therapy, which is used to treat lung infections in cystic fibrosis patients.

## MATERIALS AND METHODS

### Bacterial strains and growth conditions.

Strains (Tables 1, 3, and S4) and the HDTM library were grown in LB-Lennox or LB-Miller medium (Difco) supplemented with 100 µg/ml trimethoprim or 20 µg/ml chloramphenicol as required and incubated at 37°C with shaking. The HDTM library was grown with 0.2% rhamnose. A Biotek Synergy 2 multimode plate reader was used to provide continuous orbital shaking at 230 rpm and 37°C for growth curves in 96-well format. Cystic fibrosis sputum clinical isolates of M. abscessus were grown and maintained in LB-Lennox medium.

### Antibiotic formulations.

C109 was synthesized as previously described (12). Antibiotic working stock solutions were prepared as follows: C109, 10 mg/ml in dimethyl sulfoxide (DMSO); trimethoprim, 50 mg/ml in DMSO (Sigma); doxycycline hyclate, 25 mg/ml in H_2_O (Sigma); chloramphenicol, 20 mg/ml in ethanol (EtOH; Sigma), meropenem, 10 mg/ml in DMSO (Sigma); tobramycin sulfate, 10 mg/ml in H_2_O (Alfa Aesar); ceftazidime, 10 mg/ml in 0.1 M NaOH (Sigma); ciprofloxacin, 10 mg/ml in 0.1 M HCl (Sigma); novobiocin sodium, 10 mg/ml in H_2_O (Sigma); and piperacillin sodium salt, 10 mg/ml in H_2_O (Sigma). IPTG and L-rhamnose were obtained from Sigma.

### Antimicrobial susceptibility testing using MIC, checkerboard assay, MBIC, and MBEC.

To assess the MICs of clinical and lab strains, the broth microdilution method was employed using guidelines provided by the Clinical and Laboratory Standards Institute (59, 60), while the MIC values for the Burkholderia clinical isolates were detected using the resazurin viability stain (61). Mycobacterium inocula in cation-adjusted Mueller-Hinton broth (CAMHB; Oxoid) were prepared from growth on LB agar plates. All other inocula were prepared from overnight cultures diluted to a turbidity equal to a 0.5 McFarland standard and then further diluted to produce 5 × 10^5^ CFU/ml in each well. The initial bacterial concentrations were verified by plating for CFU on LB agar plates. Antibiotic interactions were assessed using broth microdilution checkerboard assays with inocula prepared as outlined above.

To assess the activity of C109 against biofilm formation (minimum biofilm inhibitory concentration [MBIC]) and bacterial clearance from established biofilms (minimum biofilm eradication concentration [MBEC]), a resazurin-based microplate assay was used (62). In brief, an inoculum of B. cenocepacia K56-2 in CAMHB was incubated stationary at 37°C for 4 h in 96-well format to allow substrate attachment. Planktonic cells were then washed away, and the medium was replaced with either fresh CAMHB (for the MBEC) or a C109 gradient in CAMHB (for the MBIC) and incubated again for 24 h. To determine the MBIC, C109-treated biofilms were washed and incubated stationary with CellTiter-Blue (Promega) at 37°C for 1 h. Only viable cells can reduce resazurin to the fluorescent resorufin, which was measured by excitation at 530/25 nm and emission at 590/30 nm. To determine the MBEC, day-old established biofilms were washed and exposed to a C109 gradient for a further 24 h and treated with CellTiter-Blue as described above.

### Creation of the redundant and the combined knockdown mutant library.

The detailed methodology is provided in the supplemental material. Briefly, the HDTM library was grown until early log phase, washed, resuspended in LB, and regrown until the culture reached an optical density at 600 nm (OD_600_) of 0.18. The culture was treated twice with meropenem at a final concentration of 160 μg/ml and incubated for 3 h at 37°C. Cells were washed with LB and plated on LB agar with 0.2% rhamnose and 100 μg/ml trimethoprim. After incubation at 37°C for 48 h, colonies were robotically transferred to 96-well master plates with LB and 0.2% rhamnose and 100 μg/ml trimethoprim using a Genetix QPix2 XT colony picker (Molecular Devices). After incubation at 37°C for 48 h, the master plates were robotically inoculated into 96-well plates containing LB with 100 μg/ml trimethoprim and with or without 0.2% rhamnose; after incubation, the conditional growth phenotype was assessed as 50% or less growth (by OD_600_) without rhamnose. Putative conditional growth phenotypes were validated by secondary screening under the same conditions and stored as glycerol stocks (redundant knockdown mutant library, 830 clones). To build the combined knockdown mutant library, the redundant knockdown mutant library and 134 previously obtained knockdown mutants (15) were pooled in equal amounts by OD_600_, and the pool aliquots were stored as glycerol stocks.

### Competitive fitness assay and sequencing data analysis.

The detailed methodology is provided in the supplemental material. Briefly, all the knockdown mutants were inoculated into 5 ml LB 0.05% rhamnose, with or without C109 or novobiocin, added at a concentration that inhibited 25% of K56-2 growth (IC_25_, 2.5 and 2 μg/ml, respectively). The cultures were grown for 20 generations at 37°C with shaking. Mutants recovered after growth without antibiotics are shown in Table S1. Cultures were harvested, the genomic DNA was isolated, and the Tn-seq circle method was performed as previously described (14, 16). The PCR primers used are listed in Table S5. Indexed samples were with an Illumina HiSeq 2500 system. All custom scripts used for data processing can be found at https://github.com/mdomarat/CardonaLab. Reads were normalized by total read count, and significance was assessed by calculating *P* values as per Pierce et al. (63). Log_2_(depletion) values for each mutant were calculated as the log_2_ ratio of the average normalized reads in the no-antibiotic control to those of the antibiotic-treated sample. Log_2_(depletion) values of mutants that passed the significance threshold of a *P* value of <0.05 were fit to a normal distribution (64), and candidate targets were taken as greater than two standard deviations from the mean.

### Overexpression of *dcw* proteins in the presence of C109.

The effect of *dcw*-GFP fusion protein overexpression was determined by comparing the growth curves of each of the strains in various IPTG concentrations to that of the strain harboring pCA24N-empty, the empty vector control for the ASKA library (23). An overnight culture of the appropriate strains was diluted to low OD_600_ and grown until the culture reached an OD_600_ of 0.3 to 0.6. Mild overexpression was then induced with 10 μM IPTG for 1.5 h. Cells were washed and used to inoculate a C109 gradient containing 0.01 mM IPTG. After 12 h of exposure at 37°C and shaking at 230 rpm, aliquots were plated for counting the CFU per milliliter. To facilitate interassay comparison, the CFU per milliliter for each strain was normalized to the condition without C109. These ratios were then further normalized to the strain carrying pCA24N-empty at each concentration of C109. The results were reported as abundance relative to the strain containing pCA24N-empty. A 2-fold change in abundance was taken as significant.

### Construction of CG*ftsZ* and CG*topB*.

Using the K56-2 background, an unmarked insertion of the following sequence was made between WQ49_RS12570 (BCAL3479) and WQ49_RS12575 (BCAL3480): 5′-TCTTAATTAATTTAAATCTAGACTAGTGCGGCCGCACTTGTGTATAAGAGTCATAAGAGACAG-3′. This sequence is not found in the K56-2 genome and can be used to track transposon mutants via Tn-seq. These genes encode a putative anti-sigma factor and a hypothetical protein, respectively. Previously determined criteria for the creation of a stable genetically barcoded strain were used to select the insertion site (65). Additionally, differential RNA sequencing (dRNA-seq) data for the region were examined, which found it to be transcriptionally inactive, at least in B. cenocepacia J2315 grown in biofilms (66).

The method of Flannagan et al. was used for mutant construction (67). Briefly, a 996-bp fragment including the 3′ ends of WQ49_RS12570 and WQ49_RS12575, the intergenic region, the unique transposon sequence, and KpnI and EcoRI restriction sites was synthesized (IDT). The fragment was digested with KpnI and EcoRI (NEB), ligated into pGPI-SceI to create pAH3, and then transformed into E. coli SY327. Using E. coli SY327/pRK2013 as a helper strain, pAH3 was introduced into K56-2 via triparental mating. The origin of pGPI-SceI is nonfunctional in Burkholderia spp.; therefore, exconjugants resistant to 100 µg/ml trimethoprim had pAH3 recombined into the genome. The presence and correct placement of the recombination event were verified with PCR (Qiagen). To facilitate the second recombination, pDAI-SceI, which expresses the I-SceI homing endonuclease, was introduced via triparental mating. Exconjugants sensitive to trimethoprim and resistant to 100 µg/ml tetracycline were selected, and the second recombination was verified by PCR. The mutant was cured of the plasmid by serial passaging in tetracycline-free medium and combined with the conditional growth mutant libraries in the competitive enhanced susceptibility assays.

This Tn-tagged mutant of K56-2 was then used as background for the insertion of pAH1 by homologous recombination, as described previously (2), to create CG*ftsZ*. Briefly, the 5,301 bp of *ftsZ* (WQ49_RS00110 [BCAL3457]) was PCR amplified from the K56-2 genome with 5′-NdeI and 3′-XbaI restriction sites. The fragment was ligated into pSC201 immediately downstream of the rhamnose-inducible promoter, resulting in pAH1. This plasmid was introduced into the Tn-tagged K56-2 by triparental mating, as described above. To create CG*topB*, the 5,406 bp of *topB* (WQ49_RS23460 [BCAL0462]) was amplified and ligated into pSC201 as described above, resulting in pAH5, and introduced into K56-2 as described above. Insertional mutants were verified by PCR and by rhamnose-dependent growth. WQ49_RS23460 was named the B. cenocepacia homologue of *topB* by reciprocal best-hit BLAST against the E. coli. The same method was applied for *xseB*, *ispA*, *dnaN*, *holC*, and *pepA*.

### Light and fluorescence microscopy.

For E. coli strains expressing the *dcw* protein-GFP fusions, fresh overnight cultures with 20 μg/ml chloramphenicol were subcultured the next morning with 4 µg/ml C109 or 1 μg/ml cefotaxime without chloramphenicol and IPTG and grown until mid-exponential phase (approximately 3 h). To account for the difference in doubling time, B. cenocepacia K56-2 and mutants were incubated for 6 h with 8 µg/ml C109 or with 100 µg/ml trimethoprim and 0 or 0.2% rhamnose, respectively. Prior to staining, cells were fixed with 4% formaldehyde (Sigma) in phosphate-buffered saline (PBS) at room temperature for 20 min. Cells were then washed with PBS and stained with 4′-diamidino-2-phenylindole (DAPI) (Thermo Fisher Scientific) and Nile red (Carbosynth). The cells were then immobilized on 1.5% agarose pads (Invitrogen) and visualized on an Axio Observer Z1 inverted microscope (Carl Zeiss Microscopy GmbH).

For time-lapse microscopy, overnight cultures were subcultured without IPTG or C109 and grown to mid-exponential phase. Fifteen minutes before cells were harvested, 4 µg/ml C109 was added to the culture. Cells were diluted to an OD_600_ of 0.2 and spotted on a 1.5% low-melt agarose pad (Life Technologies) made with LB and impregnated with 4 µg/ml C109. The temperature was maintained with a TempModule S control unit (Carl Zeiss Microscopy GmbH) mounted on an Axio Observer Z1 inverted microscope. At each time point, differential interference contrast (DIC) and GFP filter images were acquired. Exposure time was limited to mitigate cytotoxicity.

### Clonal growth assay of susceptibility.

The rhamnose dose-growth response and C109 inhibition curves were first determined in K56-2, CG*dcw*, and CG*ftsZ*. In 96-well plate format, the mutants were then grown in concentration of rhamnose required to produce 45% of wild-type K56-2 growth (0.04% for CG*dcw* and 0.05% for CG*ftsZ*) and in the C109 IC_10_ – IC_50._ The OD_600_ of technical triplicates was measured after 20 h. Log_2_(depletion) was calculated as the log_2_ of the average OD_600_ under the no-antibiotic condition divided by the OD_600_ in the presence of C109.

### Cloning, expression, and purification of B. cenocepacia J2315 FtsZ.

Using the primers pet28presFtsZfor (5′-ATGGGTCGCGGATCCCTGGAAGTTCTGTTCCAGGGGCCCATGGAATTCGAAATGCTGGA-3′) and pet28ftsZrev (5′-TGCGGCCGCAAGCTTTCAGTCAGCCTGCTTGCGCA-3′), *ftsZ* (BCAL3457) was amplified from B. cenocepacia J2315. PCR products were cloned into the pET-28a vector (Novagen) using the In-Fusion HD Cloning kit (TaKaRa), according to the manufacturer’s instructions. Heterologous protein production was achieved in E. coli BL21(DE3), inducing the expression with 0.5 mM IPTG overnight at 20°C. Cells were kept frozen at −80°C until needed and then thawed and resuspended in buffer (50 mM Tris-HCl, 300 mM KCl, 5 mM imidazole, and 10% glycerol [pH 8]) and sonicated. The lysates were centrifuged (50,000 × *g* for 1 h) and applied on a HisTrapFF Crude column (1 ml; GE Healthcare). The column was washed with 20 mM imidazole, and then B. cenocepacia J2315 FtsZ (BcFtsZ) was eluted with 250 mM imidazole. Proteins were dialyzed against 50 mM Tris-HCl (pH 7.5), 150 mM NaCl, 1 mM EDTA, 1 mM dithiothreitol (DTT), and 10% glycerol and then treated with PreScission protease (GE Healthcare) to remove the N-terminal histidine tag. Finally, BcFtsZ was further purified by gel filtration chromatography (HiLoad 16/60 Superdex-75 column; GE Healthcare) in 20 mM Tris-HCl (pH 7.9), 50 mM KCl, 1 mM EDTA, 2.5 mM Mg(CH_3_COO)_2_ and 10% glycerol, concentrated to 6 mg/ml, and stored at −80°C until needed.

### *In vitro* assay of BcFtsZ GTPase activity.

GTPase activity was assayed at 30°C, using a pyruvate kinase–l-lactic dehydrogenase (PK/LDH) spectrophotometric coupled assay (68). The reaction mixture was first set up to contain 50 mM HEPES (pH 7.5), 5 mM MgCl_2_, 5 mM KCl, 10 U PK/LDH, 0.25 mM NADH, 0.25 mM phosphoenolpyruvate, and 5.25 µM BcFtsZ. The assay was initiated by the addition of 0.5 mM GTP. Steady-state kinetic parameters were determined by monitoring the absorbance at 340 nm at various concentrations of GTP. The experiments were performed in triplicate, and the kinetic constants were determined by fitting the data to the Michaelis-Menten equation using Origin 8. C109 was added in concentrations ranging from 0.5 µM to 100 µM, and the inhibitory concentration that reduced the activity by half (IC_50_) was determined using Origin 8. *A*_[I]_ is the enzyme activity at inhibitor concentration [I], and *A*_[0]_ is the enzyme activity without inhibitor.

$$A_{\left[I\right]}=A_{\left[0\right]}\times \left(1-\frac{\left[I\right]}{\left[I\right]+{\text{IC}}_{50}}\right)$$

The activities of PK and LDH individually were assayed with C109, but no effect was detected, thus excluding the possibility that the molecule can exert an effect on them.

### *In vitro* BcFtsZ polymerization, using sedimentation and electron microscopy.

FtsZ polymerization was performed using a previously described sedimentation protocol (34). The reaction mixture was set up to contain 25 mM PIPES (pH 6.8), 10 mM MgCl_2_, 12 µM BcFtsZ, and 2 mM GTP or GDP. The reaction mixtures were incubated for 10 min at 30°C and 300 rpm to allow the polymerization to occur. Subsequently, samples were ultracentrifuged at 350,000 × *g* for 10 min at 25°C, and the supernatant was immediately separated from the pellet, which contains the protein polymers. The samples were analyzed by SDS-PAGE on 12% polyacrylamide gels. The *in vitro* polymerization of BcFtsZ was tested in the presence of increasing concentrations of the C109 ranging from 10 µM to 100 µM.

BcFtsZ polymers were visualized by negative-stain electron microscopy. The polymerization reactions were carried out under the same conditions described above for the sedimentation assay. After the incubation time, a small aliquot of the reaction mixture was applied onto a glow-discharged 300 mesh carbon-coated nickel grid. Subsequently, the grid was stained with a 2% uranyl-acetate solution. The grid was analyzed with a Zeiss EM900 electron microscope (Jena, Germany) operating at 80 kV.

### C. elegans infection assay.

To assess the *in vivo* antibiotic activity of C109 on K56-2 infection in C. elegans, a liquid killing assay was performed as previously described (40, 69, 70). In brief, C. elegans DH26 (from the Caenorhabditis Genetics Center) was hatched and grown on nematode growth medium (NGM) agar to the L4 stage over 48 h at 25°C by feeding on E. coli OP50. For the infection, the nematodes were washed from the plates with M9 buffer and placed onto fresh plates with B. cenocepacia K56-2 or E. coli OP50 (no-infection control) and incubated at 25°C for 16 h. The nematodes were then washed from the plate with M9 buffer, allowed to settle, rinsed with M9, and then resuspended in liquid killing medium (80% M9 and 20% NGMII). Approximately 20 to 30 nematodes in liquid killing medium were deposited into individual wells of a 96-well plate with or without antimicrobials. The wells were assessed every 24 h for 6 days under a dissecting microscope for live (S-shaped and moving) and dead (straight and not moving) nematodes.

### Hemolysis assay.

The hemolytic activity of C109 was determined as previously described (40, 70, 71), with modifications. Briefly, ovine erythrocytes (Alere) were washed thrice in PBS and resuspended in PBS to give a 20% stock. In 96-well format, a 100-μl dilution gradient of DMSO and C109 was set up, to which was added 100 μl of the 20% erythrocyte stock. The plate was incubated stationary at 37°C for 1 h. Intact erythrocytes were pelleted by centrifugation at 1,500 × *g* for 5 min, and the absorbance of the supernatant at 540 nm was measured. As a positive control, 0.1% Triton X-100 was added to the wells. Hemolysis was calculated as previously described (72). High, low, and absent hemolytic activity were defined as >40%, between 5% and 10%, and <5%, respectively (72).

### MTT assay.

Human bronchial epithelial cells (both wild type, 16HBE, or homozygous for the ΔF508 mutation in *C*FTR, CFBE41o-) were used to assess the toxicity of C109. Cells were cultured in minimal essential medium (MEM) supplemented with fetal bovine serum (10%), l-glutamine (1%), penicillin (100 U/ml), and streptomycin (100 μg/ml) and maintained at 37°C in 5% CO_2_ atmosphere. All reagents were purchased from Life Technologies. Cells were seeded at a density of 2.0 × 10^4^ cells per well in 96-well plates. After 24 h, the medium was refreshed, and cells were exposed to a C109 concentration gradient (12.5 to 100 µM) for 3 h. The wells were then gently washed with PBS and incubated with 200 μl of 0.5 mg/ml 3-(4,5 dimethylthiazol-2-yl)-2,5-diphenyltetrazolium bromide (MTT; Sigma-Aldrich) at 37°C for 3 h. The solution was removed, and the formazan crystals were dissolved by adding DMSO to the wells for 10 min, after which the absorbance at 570 nm was measured using a plate reader (EZ Read400; Biochrom).

### Data availability.

Raw reads were deposited in the NCBI Sequence Read Archive (SRA) repository under accession number SRP148709.

## Supplementary Material

Supplemental file 1

Supplemental file 2
