# Supplementary material for: Competitive Fitness of Essential Gene Knockdowns Reveals a Broad-Spectrum Antibacterial Inhibitor of the Cell Division Protein FtsZ
Source: Antimicrob Agents Chemother. 2018 Nov 26;62(12):e01231-18. doi: 10.1128/AAC.01231-18 (PMC6256756; doi:10.1128/AAC.01231-18)
Supplement: Supplemental file 2 [file zac012187678s2.pdf]

Supplementary Material

Supplementary Material and Methods

Figs. S1 to S7

Tables S2 to S5 (Table S1 is provided as a separate Excel file).

Creation of the Redundant and the Combined Knockdown Mutant Library

The initial high-density transposon mutant (HDTM) library was obtained by delivering a transposon element with an outward, rhamnose-inducible promoter with selection of transposon mutants in the presence of rhamnose (1). The HDTM library was grown in LB with 0.2% rhamnose and 100 µg/mL trimethoprim at 37°C until early log phase (OD_{600nm} ~0.13-0.18) and the cells were washed twice by centrifugation and resuspension in LB. The cells were diluted to an OD_{600nm} of 0.01 and grown in LB (without rhamnose) until the culture reached an OD_{600nm} of 0.18. Meropenem was then added to a final concentration of 160 µg/mL (5-fold the MIC for K56-2) and incubated for 3 hours at 37°C to kill actively growing cells (non-essential gene mutants) (Hogan *et al.* manuscript in preparation). The culture was then washed once, resuspended in LB, and grown for 30 minutes at 37°C. To kill remaining non-essential gene mutants the culture was treated a second time with meropenem as before. Intact cells were removed by filtration, washed off with LB, and plated on 500 cm^2 QTrays supplemented with 0.2% rhamnose and 100 µg/mL trimethoprim. QTrays were incubated at 37°C for 48 hours. Colonies were robotically transferred to 96-well plates with LB and 0.2% rhamnose and

100 µg/mL trimethoprim using a Genetix QPix2 XT colony picker (Molecular Devices). These master plates were grown stationary at 37°C for 48 hours.

For primary screening, cultures in the master plates were robotically inoculated into 96-well plates containing LB with 100 µg/mL trimethoprim and with or without 0.2% rhamnose. After 16 hours of incubation without shaking at 37°C, the conditional growth phenotype was assessed as 50% or less growth (by OD_{600nm}) without rhamnose. Putative conditional growth phenotypes were validated by secondary screening for growth with or without rhamnose as before. Mutants that passed both screens (approximately one third of the initial clones) were stocked in LB with 20% glycerol and kept at -80°C. The transposon insertion sites were determined by Tn-seq circle (1, 2). The redundant knockdown mutant library (830 clones) and 134 previously obtained knockdown mutants (3) were grown in 96-well format in LB with 0.2% rhamnose and 100 µg/mL trimethoprim at 37°C overnight. The following day, mutants were pooled in equal amounts by OD_{600nm}, forming the combined knockdown mutant library. Pool aliquots were stored in LB with 20% glycerol at -80°C until needed.

Competitive Fitness Assay and Sequencing Data Analysis

For culturing, all the knockdown mutants were inoculated into 5 mL LB to a final OD_{600nm} of 0.0025 with individual mutants at an approximate OD_{600nm} of $2.5\ X\cdot 10^{-6}$ and with or without C109 or novobiocin, added at a concentration that inhibited 25% of K56-2 growth (IC_{25}, 2.5 and 2 µg/mL, respectively). Rhamnose was added at the sensitizing concentration of 0.05%, which produced 30% to 60% of wild-type growth. The cultures

were grown for 20 hours (approximately 20 generations) at 37°C with 230 rpm shaking. Mutants that were recovered after growth without antibiotics are shown in Table S1. Wild-type *B. cenocepacia* K56-2 controls to check for 25% growth inhibition, and single mutant cultures to check for 30-60% wild-type growth were set up alongside the mutant pools and assessed by OD_{600nm} after 20 hours. Cultures were harvested, the genomic DNA was isolated, and the Tn-seq circle method were performed as previously described (1, 2). PCR primers 681, 690, 715, 717, 718, 719, 729 contain the Nextera indices and were used in appropriate pairs (Table S5). Indexed samples were pooled and sequenced at Génome Québec with an Illumina HiSeq 2500. Raw reads were deposited in the NCBI Sequence Read Archive (SRA) repository and will be publicly available after publication under accession SRP148709. All custom scripts used for data processing can be found at https://github.com/mdomarat/CardonaLab. The DNA reads were trimmed (filter_reads.py) and mapped (map_reads.py) to the contigs of the K56-2 draft genome (1). Insertion sites were then called and annotated with affected genes (identify.py). To compare the C109- or novobiocin-treated samples to the no antibiotic controls, insertion sites were merged based on position (expression.py). All insertion sites with fewer than 1000 reads in the no antibiotic controls were removed from the analysis. Reads were then normalized by total read count. Significance was assessed by calculating *P*-values as per Pierce et al. (4). Log_2(Depletion) values for each mutant was then calculated as the log_2 ratio of the average normalized reads in the no antibiotic control to those of the antibiotic-treated sample. Log_2(Depletion) values of mutants that passed the significance threshold of P-value < 0.05 were fit to a normal distribution (5)

and candidate targets were taken as greater than two standard deviations from the mean.

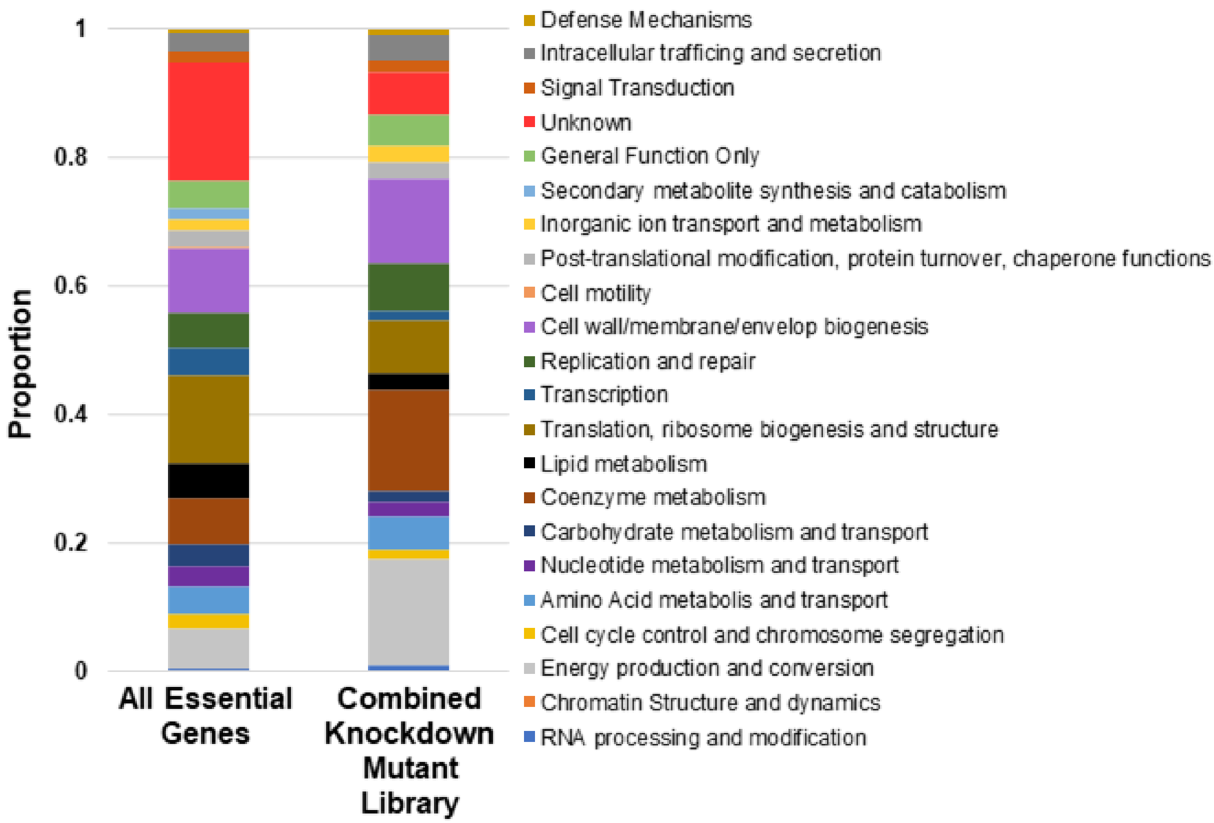

Figure S1. Essential genes in *B. cenocepacia* K56-2 and in the combined knockdown mutant library. Bars are colour-coded based on COG category. Essential genes in K56-2 are from Gislason et al. (2017).

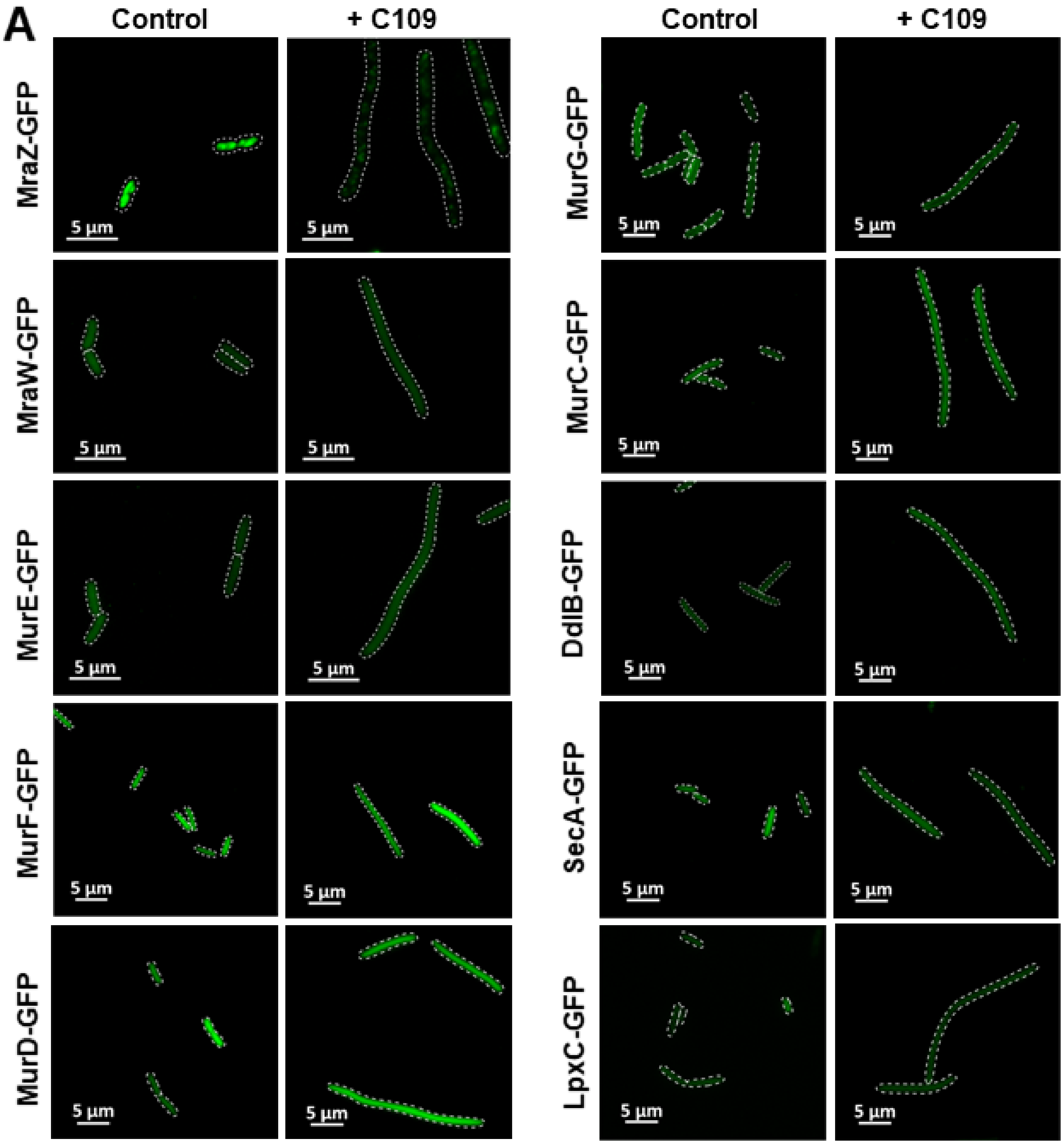
A
Control
+ C109
Control
+ C109
MraZ-GFP
MraW-GFP
MurE-GFP
MurF-GFP
MurD-GFP
MurG-GFP
MurC-GFP
DdlB-GFP
SecA-GFP
LpxC-GFP
5 µm

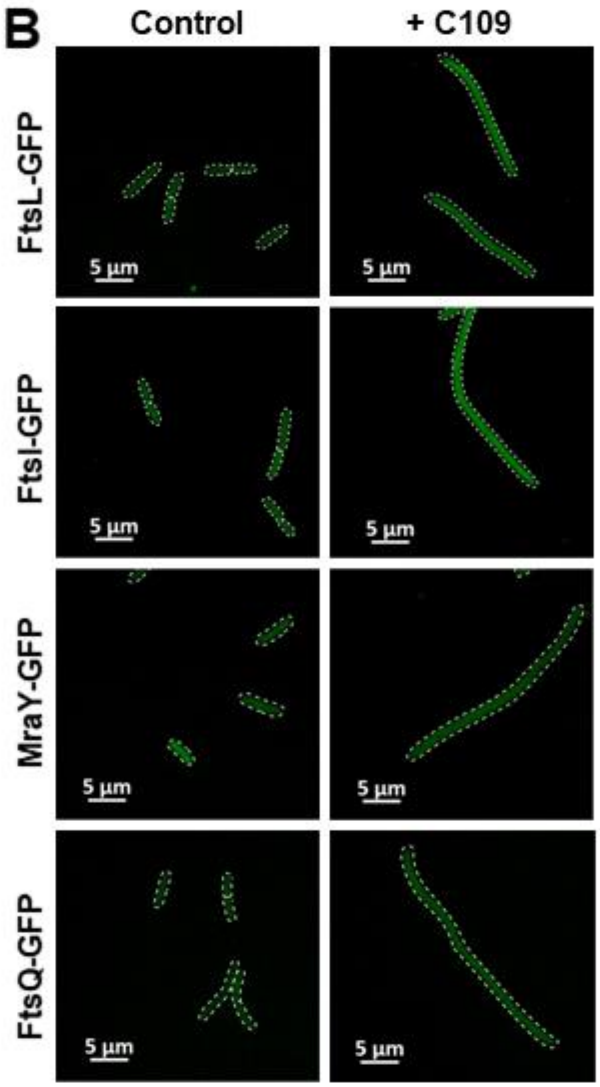

Figure S2. Treatment of the *dcw*-GFP fusion ASKA stains. The noted ASKA strains were subcultured to reach exponential phase then treated with 4 µg/mL C109 for 3 hours. Dashes denote cell boundaries. **A** Fusions that localized properly despite addition of C109. MraW, MurE, MurF, MurD, MurC, DdlB, SecA, and LpxC are primarily cytoplasmic. **B** Fusions that did not localize properly in the control condition.

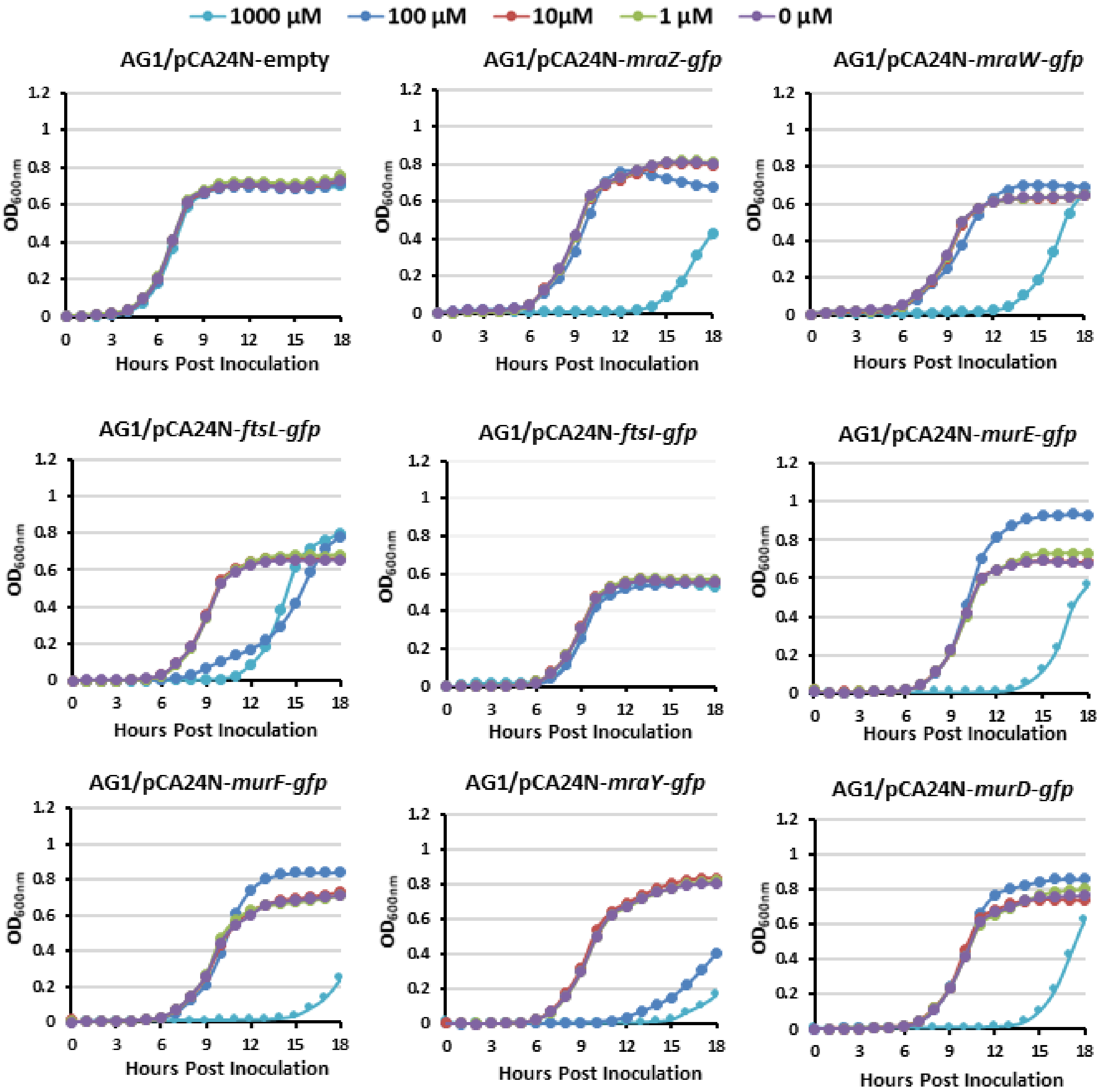
1000 µM
100 µM
10µM
1 µM
0 µM
AG1/pCA24N-empty
AG1/pCA24N-mraZ-gfp
AG1/pCA24N-mraW-gfp
AG1/pCA24N-ftsL-gfp
AG1/pCA24N-ftsI-gfp
AG1/pCA24N-murE-gfp
AG1/pCA24N-murF-gfp
AG1/pCA24N-mraY-gfp
AG1/pCA24N-murD-gfp
OD600nm
Hours Post Inoculation

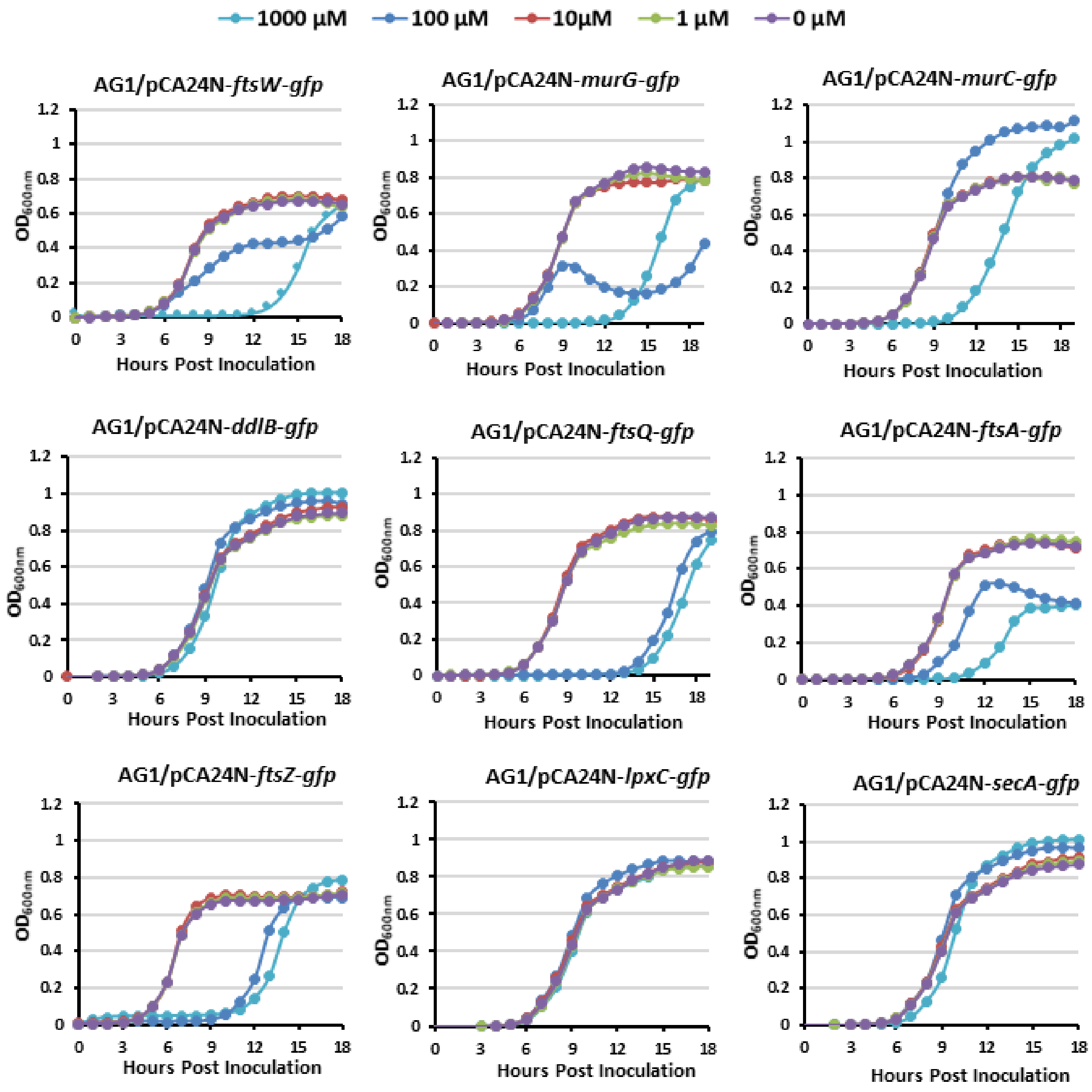

Figure S3. ASKA strains expressing *dcw* protein-GFP fusions grown in varying IPTG concentrations. Values are expressed as the means of two biological replicates.

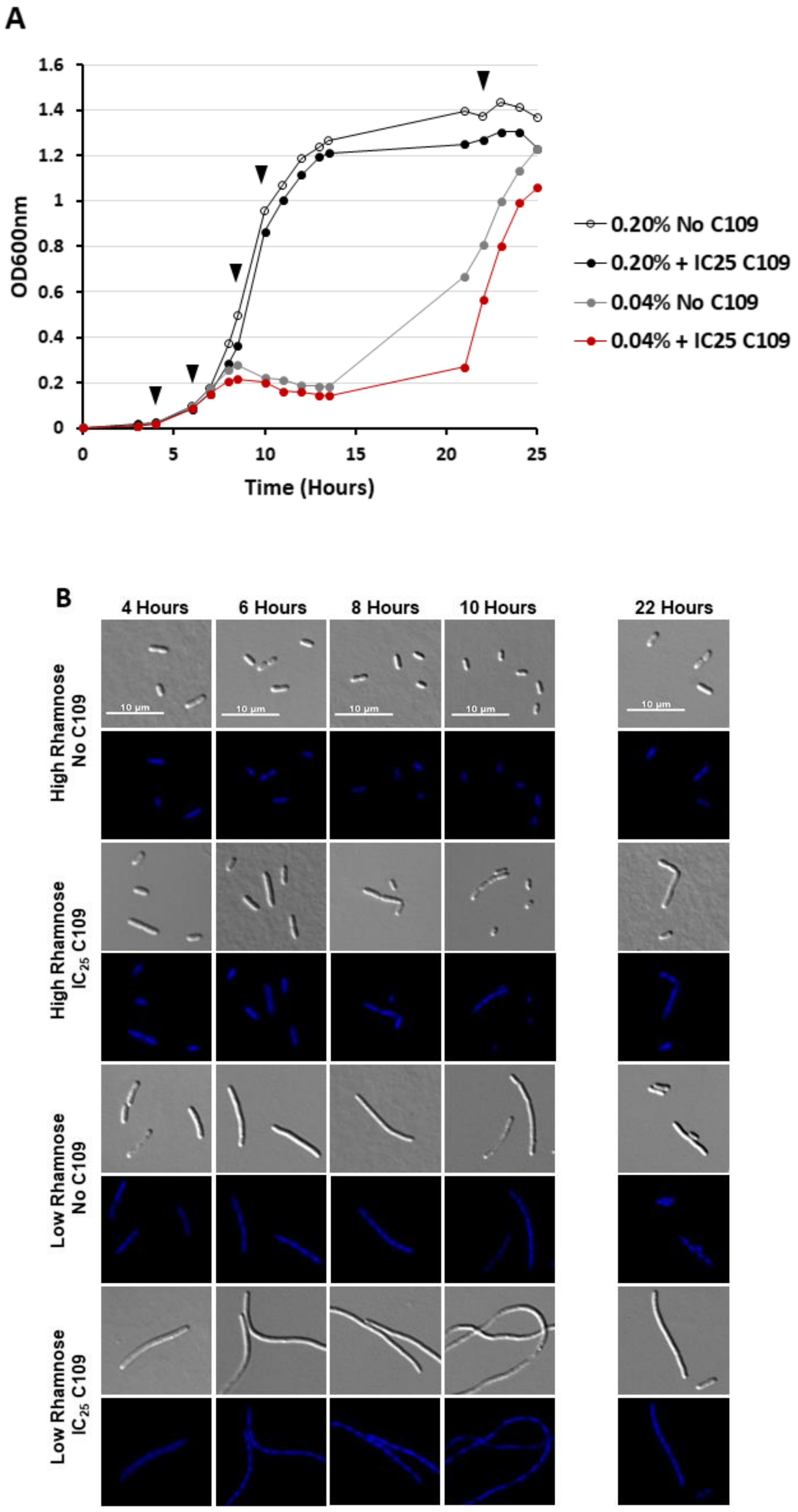
A
OD600nm
Time (Hours)
0.20% No C109
0.20% + IC25 C109
0.04% No C109
0.04% + IC25 C109
B
4 Hours
6 Hours
8 Hours
10 Hours
22 Hours
High Rhamnose No C109
High Rhamnose IC25 C109
Low Rhamnose No C109
Low Rhamnose IC25 C109
10 μm

Figure S4. Knockdown of *ftsZ* enhances the filamentous phenotype in response to C109. Cells were inoculated in medium with high (0.20%) or low (0.04%) rhamnose with or without the IC_{25} of C109. At each timepoint, the **A** OD_{600nm} was recorded and samples were processed for **B** DIC and DAPI fluorescence microscopy. Wedges in **A** indicate when samples were processed for microscopy.

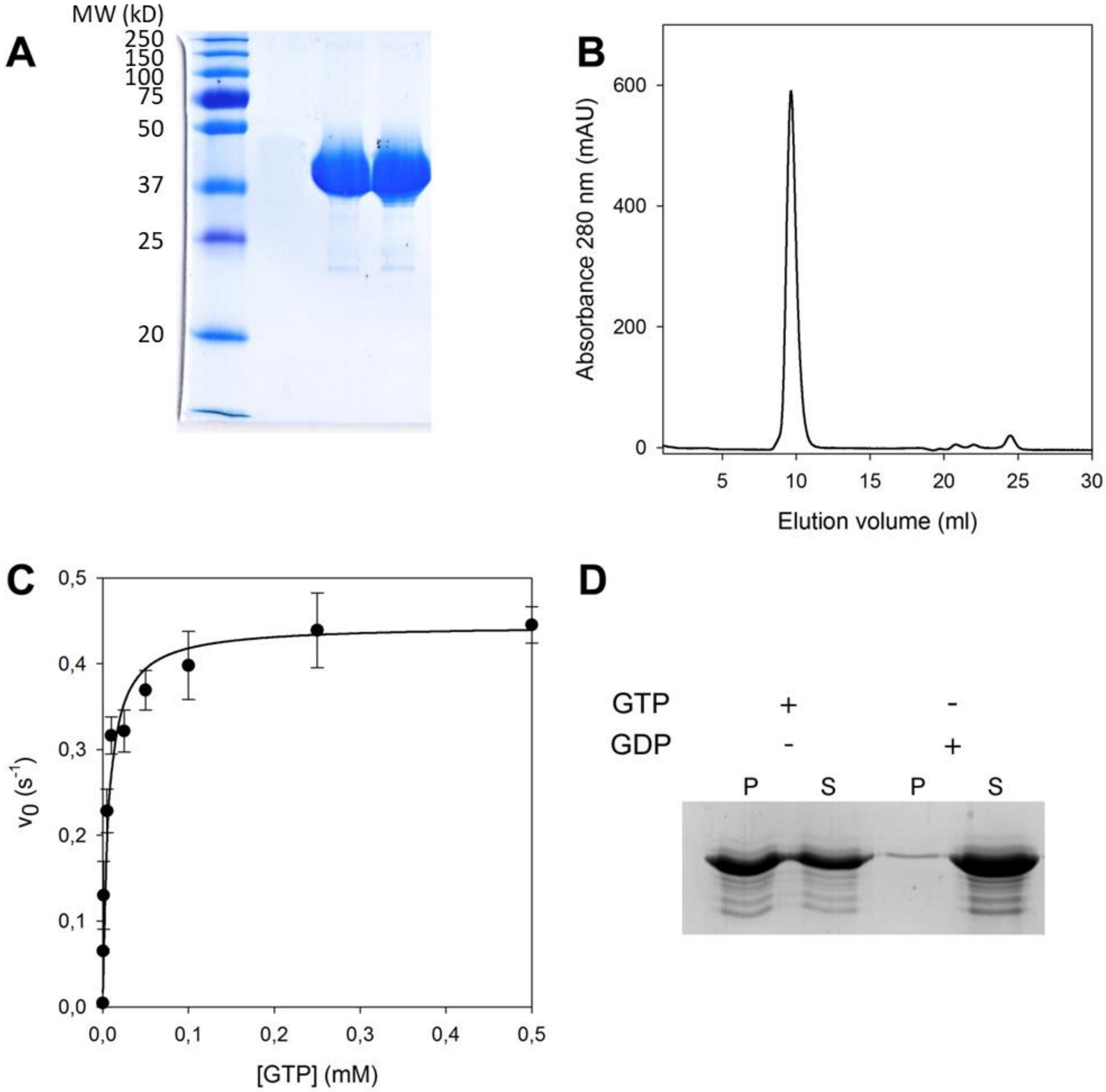

Figure S5. Biochemical characterization of the recombinant *B. cenocepacia* FtsZ. The recombinant *B. cenocepacia* FtsZ was purified to homogeneity in a soluble and monomeric protein, catalytically active and able to form polymers. **A** SDS-PAGE of the purified FtsZ. **B** Analytical gel filtration profile of the protein on a Superdex 75 column. **C** Steady state kinetic analysis of FtsZ activity as a function of GTP concentration. **D** SDS-PAGE of the sedimentation of FtsZ tubules, P: pellet fraction, S: sedimentation fraction.

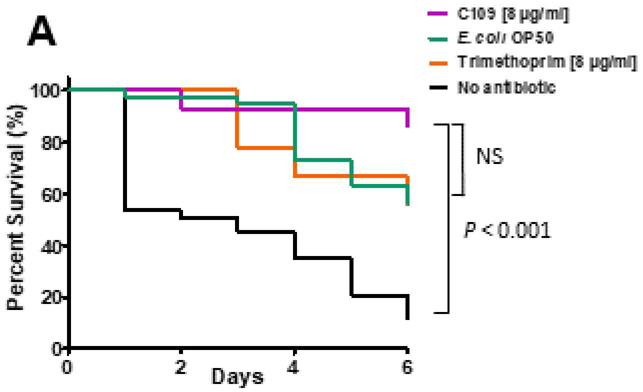

B

2-Day Survival from Panel A	
Fed with *E. coli* OP50	98% (NS)
No Antibiotic	51% ($P < 0.001$)
Trimethoprim (8 µg/mL)	100% (NS)
C109 (8 µg/mL)	93%

Figure S6. C109 rescues *C. elegans* from *B. cenocepacia* infection and is not toxic to the nematodes. **A** *C. elegans* nematodes were infected with strain K56-2 and treated with 8 µg/mL of C109 or trimethoprim and observed over 6 days. **B** Survival of *C. elegans* at 2-days post-infection. Significance from the C109-treated sample was determined by Mantel-Cox log-rank test. Values are means from three biological replicates. NS, not significant.

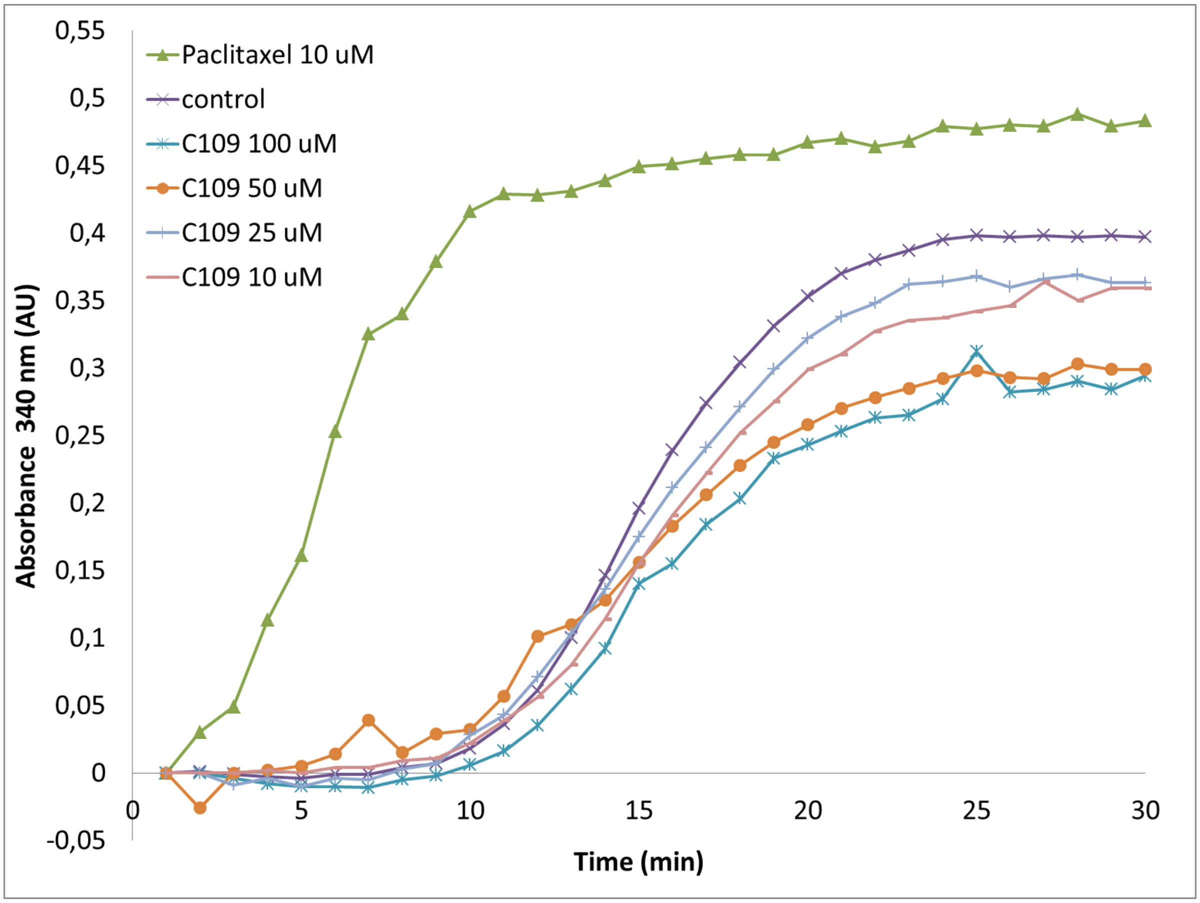

Figure S7. C109 does not affect tubulin polymerization. Set amounts of bovine tubulin and GTP were incubated with paclitaxel or increasing concentrations of C109. Light scattering at 340 nm was measured every minute.

Table S2. Morphological characteristics of the mutants hypersusceptible to C109

	**Morphology Characteristic** (% of total cells)				
**Mutant or Treatment**	Filamentation	Enlargement*	Shortening	Bending	Lysis (earliest occurrence)
C109	+ (91)	-	-	-	+ (3 hr)
CG*dcw*	+ (100)	-	-	-	+ (24 hr)
CG*ftsZ*	+ (100)	-	-	-	+ (24 hr)
CG*pepA-holC*	-	+ (87)	-	-	+ (6 hr)
CG*holC*	-	+ (82)	-	-	+ (6 hr)
CG*topB*	-	+ (78)	-	-	+ (6 hr)
CG*xseB-ispA-dxs*	-	-	+ (95)	-	-
CG*ispA-dxs*	+ (100)	-	-	+ (88)	+ (6 hr)
CG*dnaN*	-	-	-	-	+ (6 hr)

*Enlargement is defined as an increase in cell length and width

Table S3. Cytotoxic properties of C109 reported in uninfected *C. elegans,* human cells, and for ovine erythrocytes.

C. elegans	
$SURV_{100}$	128 µg/mL
$SURV_{100}$/MIC	16
6-Day Tolerance at 128 µg/mL C109	79%
**Mammalian Cells**	
Erythrocyte Lysis at 128 µg/mL	3.1%
TC_{50} (16HBE)	75 µM (21.4 µg/mL)
TC_{50} (CFBE41o-)	75 µM

All values are reported as the mean of three biological replicates

Table S4. Bacterial strains and plasmids used in this work.

Strain or plasmid	Features	Source
Burkholderia. cenocepacia CG*dcw*	WQ49_RS00035-WQ49_RS00040 (BCAL3471-3472)::pRB-rham	(3)
B. cenocepacia CG*dnaN*	WQ49_RS23255 (BCAL0422)::pRB-rham	(3)
B. cenocepacia CG*ftsZ*	WQ49_RS12570 - WQ49_RS12575 (BCAL3479-3480)::Tn-Tag *ftsZ*::pAH1 in K56-2 background	This study
B. cenocepacia CG*holC*	WQ49_RS26825 (BCAL2676)::pRB-rham	(3)
B. cenocepacia CG*ispA-dxs*	WQ49_RS16930 (BCAM0910)::pRB-rham	(3)
B. cenocepacia CG*pepA-holC*	WQ49_RS26820 - WQ49_RS26825 (BCAL2677-2676)::pRB-rham	(3)
B. cenocepacia CG*topB*	WQ49_RS23465 - WQ49_RS23460 (BCAL0463-BCAL0462)::pAH5	This study
B. cenocepacia CG*xseB-ispA-dxs*	WQ49_RS16935 (BCAM0909)::pRB-rham	(3)
E. coli AG1	*recA1 endA1 gyrA96 thi-1 hsdR17*(rK^-, mK^+) *supE44 relA1*	Dr. Eric Brown
E. coli BL21(DE3)	F^- *ompT gal dcm lon* $hsdS_B(r_B^- m_B^-)$ λ(DE3 [*lacI lacUV5-T7p07 ind1 sam7 nin5*]) $[malB^+]_{K-12}(\lambda^S)$	Pavia laboratory collection
E. coli DH5α	F^- Φ80*lac*ZΔM15 Δ(*lac*ZYA-*arg*F) U169 *rec*A1 *end*A1 *hsd*R17 (rK^-, mK^+) *pho*A *sup*E44 λ^- *thi*-1 *gyr*A96 *rel*A1	Pavia laboratory collection
E. coli SY327	F^- *araD* Δ(*lac-proAB*) *argE*(Am) *recA56* Rifr *nalA* λ*pir*	(6)

Staphylococcus aureus ATCC 29213	Methicillin-sensitive reference strain	ATCC
S. aureus CF 225	MRSA (SCC*mec* IV)	Dr. M. Mollerach (7)
pAH1	Derivative of pSC201 with 5' region of *ftsZ* (BCAL3457) downstream of the rhamnose-inducible promoter	This study
pAH3	Derivative of pGPI-*Sce*I with a cloned region of the Tn tag<br>flanked by 3' and intergenic regions of BCAL3479-3480	This study
pAH5	Derivative of pSC201 with 5' region of *topB* (BCAL0462) downstream of the rhamnose-inducible promoter	This study
pCA24N-*gfp*	ori_{colE1} Chl^r *$lacI^q$* PT5-lac *gene-gfp*	(8)
pDAI-*Sce*I	ori_{pBBR1} Tet^r mob^+ P*dhfr I-SceI*	(9)
pET-28a	ori_{pBR322} ; Kan^r; *lacI*	Novagen
pGPI-*Sce*I	ori_{R6K} Tmp^r mob^+ carries I-SceI cut site	(9)
pRB-rham	pSCrhaboutgfp derivative (10) ori_{R6K} *dhfr rhaR rhaS* P*rhaB*	(3)
pRK2013	ori_{colE1} RK2 derivative Kan^r mob^+ tra^+	(11)
pSC201	ori_{R6K} *rhaR rhaS* P*rhaB dhfr*	(12)

Table S5. Oligonucleotides used for Tn-Seq Circle.

Oligonucleotide	Sequence (5'-3')
681	CAAGCAGAAGACGGCATACGAGATTCGCCTTAGTCTCGTGG GCTCGGAGATGTGTATAAGAGACAG
690	AATGATACGGCGACCACCGAGATCTACACTAGATCGCTCGTC GGCAGCGTCAGATGTGTATAAGAGACAGNNNNAATCTAGACT AGTGCGGCC
715	CAAGCAGAAGACGGCATACGAGATCTAGTACGGTCTCGTGG GCTCGGAGATGTGTATAAGAGACAG
717	CAAGCAGAAGACGGCATACGAGATGCTCAGGAGTCTCGTGG GCTCGGAGATGTGTATAAGAGACAG
718	CAAGCAGAAGACGGCATACGAGATAGGAGTCCGTCTCGTGG GCTCGGAGATGTGTATAAGAGACAG
719	AATGATACGGCGACCACCGAGATCTACACCTCTCTATTCGTC GGCAGCGTCAGATGTGTATAAGAGACAGNNNNAATCTAGACT AGTGCGGCC
729	CAAGCAGAAGACGGCATACGAGATCATGCCTAGTCTCGTGG GCTCGGAGATGTGTATAAGAGACAG

References:

1. Gislason AS, Turner K, Domaratzki M, Cardona ST. 2017. Comparative analysis of the *Burkholderia cenocepacia* K56-2 essential genome reveals cell envelope functions that are uniquely required for survival in species of the genus *Burkholderia*. Microb Genomics 3.

2. Gallagher LA, Shendure J, Manoil C. 2011. Genome-scale identification of resistance functions in *Pseudomonas aeruginosa* using Tn-seq. mBio 2:e00315-10.

3. Bloodworth RAM, Gislason AS, Cardona ST. 2013. Burkholderia cenocepacia conditional growth mutant library created by random promoter replacement of essential genes. MicrobiologyOpen 2:243–258.

4. Pierce SE, Davis RW, Nislow C, Giaever G. 2007. Genome-wide analysis of barcoded *Saccharomyces cerevisiae* gene-deletion mutants in pooled cultures. Nat Protoc 2:2958–2974.

5. Delignette-Muller M, Dutang C. 2015. fitdistrplus: an R package for fitting distributions. J Stat Soft 64.

6. Miller VL, Mekalanos JJ. 1988. A novel suicide vector and its use in construction of insertion mutations: osmoregulation of outer membrane proteins and virulence determinants in *Vibrio cholerae* requires *toxR*. J Bacteriol 170:2575–2583.

7. Pena Amaya P, Haim MS, Fernández S, Di Gregorio S, Teper A, Vázquez M, Lubovich S, Galanternik L, Mollerach M. 2017. Molecular Epidemiology of

Methicillin-Resistant *Staphylococcus aureus* in Cystic Fibrosis Patients from Argentina. Microb Drug Resist.

8. Kitagawa M, Ara T, Arifuzzaman M, Ioka-Nakamichi T, Inamoto E, Toyonaga H, Mori H. 2005. Complete set of ORF clones of *Escherichia coli* ASKA library (a complete set of *E. coli* K-12 ORF archive): unique resources for biological research. DNA Res Int J Rapid Publ Rep Genes Genomes 12:291–299.

9. Flannagan RS, Linn T, Valvano MA. 2008. A system for the construction of targeted unmarked gene deletions in the genus *Burkholderia*. Environ Microbiol 10:1652–1660.

10. Cardona ST, Mueller C, Valvano MA. 2006. Identification of essential operons in *Burkholderia cenocepacia* with a rhamnose inducible promoter. Appl Environ Microbiol 72:2547–2555.

11. Figurski DH, Helinski DR. 1979. Replication of an origin-containing derivative of plasmid RK2 dependent on a plasmid function provided in trans. Proc Natl Acad Sci U S A 76:1648–1652.

12. Ortega XP, Cardona ST, Brown AR, Loutet SA, Flannagan RS, Campopiano DJ, Govan JR, Valvano MA. 2007. A putative gene cluster for aminoarabinose biosynthesis is essential for *Burkholderia cenocepacia* viability. J Bacteriol 189:3639–3644.
